# Modeling of Miniemulsion Polymerization of Styrene with Macro-RAFT Agents to Theoretically Compare Slow Fragmentation, Ideal Exchange and Cross-Termination Cases

**DOI:** 10.3390/polym11020320

**Published:** 2019-02-13

**Authors:** Dries J.G. Devlaminck, Paul H.M. Van Steenberge, Marie-Françoise Reyniers, Dagmar R. D’hooge

**Affiliations:** 1 Laboratory for Chemical Technology (LCT), Ghent University, Technologiepark 914, B-9052 Ghent, Belgium; Dries.Devlaminck@UGent.be (D.J.G.D.); MarieFrancoise.Reyniers@UGent.be (M.-F.R.); Dagmar.Dhooge@UGent.be (D.R.D.); 2 Centre for Textile Science and Engineering, Ghent University, Technologiepark 907, B-9052 Ghent, Belgium

**Keywords:** RAFT, miniemulsion, Smith-Ewart modelling

## Abstract

A 5-dimensional Smith-Ewart based model is developed to understand differences for reversible addition-fragmentation chain transfer (RAFT) miniemulsion polymerization with theoretical agents mimicking cases of slow fragmentation, cross-termination, and ideal exchange while accounting for chain length and monomer conversion dependencies due to diffusional limitations. The focus is on styrene as a monomer, a water soluble initiator, and a macro-RAFT agent to avoid exit/entry of the RAFT leaving group radical. It is shown that with a too low RAFT fragmentation rate coefficient it is generally not afforded to consider zero-one kinetics (for the related intermediate radical type) and that with significant RAFT cross-termination the dead polymer product is dominantly originating from the RAFT intermediate radical. To allow the identification of the nature of the RAFT retardation it is recommended to experimentally investigate in the future the impact of the average particle size (*d_p_*) on both the monomer conversion profile and the average polymer properties for a sufficiently broad *d_p_* range, ideally including the bulk limit. With decreasing particle size both a slow RAFT fragmentation and a fast RAFT cross-termination result in a stronger segregation and thus rate acceleration. The particle size dependency is different, allowing further differentiation based on the variation of the dispersity and end-group functionality. Significant RAFT cross-termination is specifically associated with a strong dispersity increase at higher average particle sizes. Only with an ideal exchange it is afforded in the modeling to avoid the explicit calculation of the RAFT intermediate concentration evolution.

## 1. Introduction

Free radical polymerization (FRP) is currently one of the most commonly applied industrial polymerization techniques, due to its relative insensitivity to impurities, compatibility with a wide range of monomers, and the possibility to use multiple reaction media including bulk, solution, suspension, and emulsion [1,2,3,4,5,6,7,8,9,10]. A drawback is the limited structural control at the molecular level, with a high dispersity (>1.5) and a restricted functionality degree in view of the synthesis of complex well-defined macromolecular architectures. To overcome this drawback, reversible deactivation radical polymerization (RDRP) or controlled radical polymerization (CRP) techniques have been developed, allowing to regulate the molecular structure of individual polymer chains and design novel molecular architectures such as block, star, comb-like, and dendritic (co)polymers inaccessible by FRP [2,4,5,7,11,12,13,14,15,16].

Despite that several RDRP techniques have been proposed over the last decennia, reversible addition-fragmentation chain transfer (RAFT) polymerization is one of the most promising ones due to its direct resemblance to FRP regarding the order of magnitude of the polymerization rate [17,18,19]. In view of prospective industrial operation, RAFT polymerization in a dispersed medium with water as the continuous phase is preferred, as it reduces the risks of overheating and runaway as a result of the highly exothermic nature of radical polymerization [2,20,21,22,23,24,25]. High monomer conversions can also be targeted without the increasing viscosity becoming a very critical issue as in bulk polymerization, which minimizes potential problems with residual monomer removal [26,27]. Moreover, for well-chosen (average) particle sizes the kinetics are influenced by compartmentalization as reactants are confined within discrete spaces with active particles even possessing only one radical so that the livingness of the RAFT polymerization can be increased compared to the bulk RAFT process [28,29,30,31,32,33,34,35,36].

**Figure 1:**
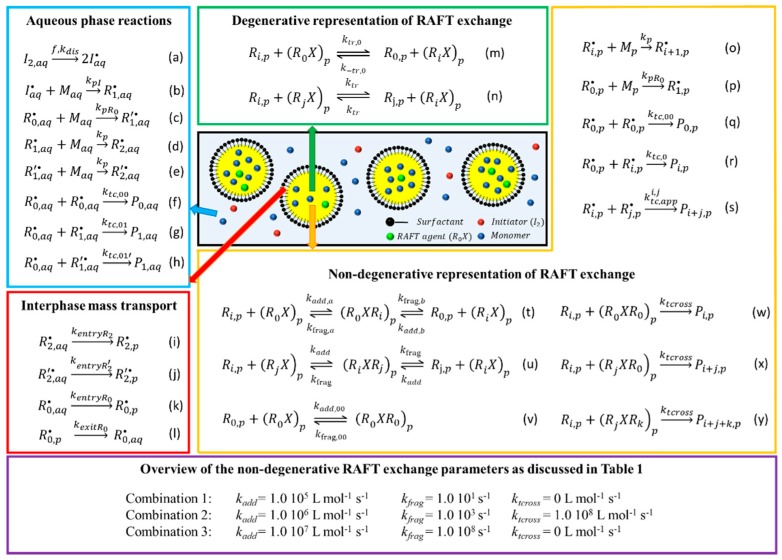
Representation of the reversible addition-fragmentation chain transfer (RAFT) miniemulsion process (black box: initialization with water soluble initiator and oil soluble initial RAFT agent) and an overview of the main reactions and interphase mass transfers, with non-degenerative defined in this work as the case in which for modeling one needs to calculate explicitly the intermediates concentrations. A subdivision is made between the reactions occurring in the aqueous phase (blue box, reaction (**a**)–(**h**)), in the organic phase (orange box, reaction (**o**)–(**y**)), and interphase mass transport phenomena (red box, (**i**)–(**l**); selected number of phenomena). For an ideal RAFT agent, the reaction sequences (**t**),(**u**),(**v**) in the orange box can be formally simplified to the “degenerative” exchange events (**m**) and (**n**) (green box; no RAFT-cross termination; reaction (**w**)–(**y**)). Conventional termination is only shown for the combination mode (**w**) as this is dominant for styrene, the monomer investigated in this work; no mass transfer for RAFT intermediate radicals, as they are likely too hydrophobic due to their size; in the present work focus is on three type of parameters combinations to theoretically reflect limiting cases (Purple box and Table 1).

A RAFT emulsion polymerization with an idealized initial state is RAFT miniemulsion polymerization (Figure 1; black box; special case of water as opposed to oil soluble initiator and oil soluble initial RAFT agent) in which the polymerization occurs in kinetically stable monomer droplets (50–500 nm) realized by high shear homogenization or ultrasonification, making aqueous phase transport of hydrophobic RAFT agent, as in conventional (macro)emulsion, not an issue [4,21,34,37,38,39,40]. Under well-defined conditions, droplet coalescence or aggregation, monomer diffusion can be restricted and, hence, the initial monomer droplet size distribution is more or less the particle size distribution [41]. A challenge remains to control the reaction rates, taking into account that radicals (besides monomer) can undergo mass transfer due to the heterogeneous nature of the process, as displayed in Figure 1 with reactions in the aqueous phase in the blue box, reactions in the organic phase in the orange box, and phase-transfer events in the red box. 

As shown Figure 1, the water soluble initiator (*I_*2*,aq_*) forms initiator fragments (*I_aq_*) that subsequently add to monomer (*M*) until oligomeric species are formed that are too hydrophobic (e.g., critical chain length for styrene, monomer in the present work: 2) to remain in the aqueous phase so that entry (interphase mass transport (i) in Figure 1) into a droplet/particle occurs. There the oligomers can grow to ‘true’ macroradicals (*R_i_*; i: chain length), which in contrast to FRP do not dominantly terminate to create dead polymer species (*P*) but can also add to the initial RAFT chain transfer agent (CTA, *R_*0*_X*), forming a *first* RAFT intermediate radical type (*R_i_XR_*0*_*, reaction (t) in Figure 1). Due to the instability of this adduct-radical, fragmentation occurs resulting in either the starting compounds (*k_frag,a_*) or a dormant macrospecies (*R_i_X*) and a RAFT CTA derived leaving group radical (*R_0_*) (*k_frag,b_*). The latter radical is capable of re-initiating the polymerization (*k_p,R0_*, reaction (s) in Figure 1) but can also undergo mass transfer to the aqueous phase (event (l) in Figure 1). Furthermore, addition of a macroradical to a dormant macrospecies leads to the formation of a *second* type of RAFT intermediate radical characterized by two arms of in general two different lengths (*R_i_XR_j_*, reaction (u) in Figure 1). Its fragmentation (*k_frag_*) can result in the reactivation of the dormant macrospecies while temporarily deactivating the original growing macroradical. If the resulting exchange/transfer of the radical function between growing and dormant macrospecies occurs fast enough, concurrent growth of all the dormant chains can be achieved. Note that a *third* type of RAFT intermediate radical (*R_*0*_XR_*0*_*) can be present in the particles, formed by the addition of the leaving group radical *R*_0_ to the initial RAFT CTA *R_*0*_X* (reaction (v) in Figure 1). Hence, five types of radicals (*R_*0*_XR_*0*,p_*, *R_i_XR_*0*,p_*, *R_i_XR_j,p_*, *R_*0*,p_* and *R_i,p_*) can exist in the particles. Considering also the aqueous phase (and the maximal chain lengths) 6 more radicals (*I_aq_*, *R(‘)_1,aq_*, *R(‘)_*2*,aq_*, and *R_*0*,aq_*) exist, ignoring the exit/entry of RAFT intermediate radicals as they are in most cases too hydrophobic. 

Under ideal RAFT conditions the consecutive/competitive RAFT additions/fragmentations (reactions (t)–(v) in the orange box of Figure 1) can be simplified to the so-called degenerative transfer mechanism (“reactions/exchanges” (m) and (n) in the green box in Figure 1). On an overall basis “direct” chain transfer is formally obtained consistent with the conventional interpretation in radical polymerization. This implies that no-RAFT cross-termination (reaction (w)–(y) in Figure 1) and no slow RAFT fragmentation is taking place so that the intermediate radical species concentrations (*R_*0*_XR_*0*,p_*, *R_*0*_XR_i,p_* and *R_i_XR_j,p_*) do not need to be explicitly calculated for modeling purposes as the pseudo steady-state approximation (PSSA) can be applied [19,42,43,44,45]. The corresponding transfer “rate coefficients” (*k_tr,*0*_*, *k_-tr,*0*_* and *k_tr_*), as defined based on the (conventional) radical and dormant species concentrations, are a function of the elementary RAFT addition and fragmentation parameters:

(1) $$k_{tr,0}=k_{add,0,a}\frac{k_{frag,0,b}}{k_{frag,0,a}+k_{frag,0,b}}$$

(2) $$k_{-tr,0}=k_{add,0,b}\frac{k_{frag,0,a}}{k_{frag,0,a}+k_{frag,0,b}}$$

(3) $$k_{tr}=\frac{k_{add}}{2}$$

with in the last equation, for simplicity, chain length dependencies neglected, although the relevance of this assumption needs still further investigation and is probably RAFT specific [46,47]. Intrinsically this assumption can be realistic [47] but at higher monomer conversion the RAFT exchanges can be influenced by diffusional limitations [19,48,49,50,51,52,53,54]. Hence, apparent exchange kinetics can be operative similar to diffusional limitations on termination, inducing the well-known gel-effect [1,34,55,56,57].

A research question that arises is which tools are most suited to test if a simplified degenerative RAFT mechanism (with upon modeling no explicit calculation needed for the concentration of the intermediate) can be applied or if a detailed mechanism (thus with an explicit calculation of the concentration of the RAFT intermediate) needs to be considered. Note that for convenience in the present work the latter mechanism is defined as “non-degenerative”. Most experimental focus has been on the bulk monomer conversion profile in which the RAFT polymerization case is compared with the corresponding FRP case (same initial conditions except no initial RAFT agent), with a (strong) rate retardation associated with the need of a non-degenerative mechanism. Barner-Kowollik et al. [58] hypothesized that the rate retardation could be explained by a low RAFT fragmentation rate resulting in an accumulation of non-propagating RAFT intermediate species. In contrast, Monteiro et al. [59] attributed the retardation to RAFT cross-termination reactions involving conventional and intermediate radicals, resulting—even with high fragmentation rates—in the formation of three and four armed dead species. Although, which explanation is most plausible is still under debate [45,60,61,62,63,64], even after a large number of both experimental [58,59,60,65,66,67,68,69,70,71,72,73,74,75,76,77,78] and theoretical studies [48,64,79,80,81,82,83,84,85,86,87,88,89]. Interestingly, recent research has indicated that bulk RAFT rate retardation can even occur under the validity of the degenerative mechanism [48,90,91]. As lower chain length polymer chains are formed in a well-defined homogeneous RAFT polymerization compared to FRP process the gel-effect is less established in the former so that the conversion profile is less steep.

Tobita et al. [92,93,94] suggested that miniemulsion RAFT polymerization with macro-RAFT agents is interesting to study the nature of rate retardation leading to a non-degenerative RAFT mechanism. The use of macro-RAFT agents neglect a first approximation of the exit/entry phenomena of *R*_0_ (mass transfer events (k) and (l) in the red box in Figure 1), which is known to induce additional retardation [36,95,96,97,98]. Still the kinetics are influenced by compartmentalization with the bimolecular RAFT cross-termination rate expected to decrease with decreasing (average) particle size, whereas the particle size should not have an influence on the RAFT fragmentation rate, at least for a given RAFT intermediate concentration. For RAFT miniemulsion polymerization of styrene mediated by polystyryl dithiobenzoate, this aspect was exploited by Suzuki et al. [93,99] who showed with intrinsic kinetic Monte Carlo simulations that the miniemulsion polymerization rate and thus monomer conversion increases with decreasing droplet size, agreeing with the theoretical bulk predictions of the RAFT cross-termination model for the investigated system. 

Despite that the investigation of RAFT in (mini)emulsion polymerization can be advantageous to investigate the plausibility of the chosen retardation model, a detailed kinetic study over a wide range of theoretically relevant RAFT addition, fragmentation, and cross-termination rate coefficients as a function of the average particle size is still lacking. Most kinetic modeling studies on RAFT (mini)emulsion polymerization are simplified [23,32,100,101,102,103,104,105,106,107,108,109,110,111,112] with the common assumption [23,103,104,110,112] of a zero-one system, hence, either droplets/particles contain one or no radical at all. For example, Altarawneh et al. [103,104] used a zero-one model to describe the RAFT emulsion polymerization of styrene mediated by *O*-ethylxanthyl ethyl propionate. The zero-one model implied that instantaneous termination occurs if a radical enters a particle containing a conventional or intermediate radical. Similarly, Luo et al. [105] showed that for the miniemulsion polymerization of styrene with styrene oligomers of 1-phenylethylphenyl dithioacetate (PS-PEPDTA) and 2-cyranoprop-2-yl dithiobenzoate (PS-CPDB) as RAFT agents, a zero-one model assuming cross-termination can be used to describe the experimental data. Li et al. [106] investigated the copolymerization of styrene and butyl acrylate in miniemulsion using 3-benzyltrithiocarbonyl propionic acid (BCPA) as the initial RAFT agent, taking into account RAFT cross-termination but no insights on the conflicting rate retardation models were given. Assuming a priori the validity of a zero-one system is not recommended. In the recent work of Devlaminck et al. [98] it was shown that for a degenerative RAFT system (polymerization of methyl methacrylate (MMA) with cyanoprop-2-yl dithiobenzoate (CPDB, *R_*0*_X*) and potassium persulfate (KPS, *I*_2_); [MMA]_0_/[CPDB]_0_/[KPS]_0_ = 590/1/0.65; *d_p_* = 100 nm) at higher monomer conversions particles with several radicals can exist due to the diffusional limitation on termination and additionally RAFT exchange in case the (average) chain length is sufficiently high.

In this work, “non-degenerative” and “degenerative” RAFT miniemulsion polymerization are theoretically compared for a wide range of RAFT exchange kinetic parameters formally aiming at limiting cases, focusing both on the monomer/initial RAFT agent conversion profile and the average polymer properties, which is rarely done. This comparison is made by means of a 5-dimensional Smith-Ewart based model, allowing the mapping of all radical types and numbers per particle, starting from the code developments in previous work on RAFT polymerization with ideal exchange [98]. It is shown that the nature of the RAFT exchange process affects the limits for the maximal radical type numbers and is dependent on the (average) particle size, with the most detailed differentiation possible upon the joint consideration of the trends for the polymerization rate and the control over average polymer properties, which should facilitate future experimental research.

## 2. Modeling Methodology

In this work, a multi-scale Smith-Ewart based modeling methodology, as recently introduced for degenerative RAFT polymerization [98], is extended to describe degenerative and non-degenerative RAFT miniemulsion polymerization of styrene (Figure 1) at 343 K. 

### 2.1. Reactions, Rate Coefficients, and Mass Transfer Parameters

The RAFT miniemulsion of styrene mediated by an oligomeric polystyrene-RAFT agent (PS-X) and initiated by potassium persulfate (KPS) as radical initiator at 343 K is theoretically investigated for several RAFT kinetic parameter combinations. Each combination is investigated considering both the simplified “degenerative (DM)” and the detailed “non-degenerative (NDM)” RAFT polymerization mechanism (*cf.* definitions in Figure 1). Three types of RAFT parameter combinations are investigated (Comb 1–Comb 3), all with a high RAFT addition rate coefficient. Firstly, Comb 1, similar to the slow fragmentation model (SFM) [58,64,99], is characterized by a high RAFT addition and a low fragmentation rate coefficient in the absence of RAFT cross-termination. Secondly, Comb 2, similar to the intermediate termination model (ITM) described in literature [59,80,99,118,119], is characterized by a high RAFT addition and cross-termination rate coefficient and a medium RAFT fragmentation rate coefficient. Thirdly, Comb 3 is characterized by a high RAFT addition and fragmentation rate coefficient with a kinetically insignificant RAFT cross termination so that the rate coefficient can be given a value of 0 mol L^−1^·s^−1^. This parameter combination mimics an ideal RAFT agent, as confirmed in the Supporting Information (Section S4). 

The use of an oligomeric RAFT agent allows neglecting interphase mass transport (entry/exit) of the RAFT leaving group (*R*_0_), which is known to have a significant impact on the polymerization kinetics at low monomer conversion [98]. Nevertheless, a differentiation is made between the initial oligomeric RAFT agent and the polymeric dormant species in order to isolate the RAFT initiation stage from the overall kinetics. For simplicity, the average particle diameter *d_p_* and the average particle volume *v_p_* are considered constant throughout the entire polymerization process, in agreement with other modeling studies [105,107,120]. 

Table 1 gives an overview of the reactions considered, with the reactions necessary to investigate the simplified degenerative and the detailed non-degenerative RAFT model also mentioned. The water soluble KPS radical initiator (*I*_2_) decomposes only in the aqueous phase. The formed radical anion (*I*) propagates until an oligomeric species with a critical chain length of 2 is formed after which only entry in the particles can occur (mass transfer event in Table 1). Entry of smaller oligomeric species and the radical anion is neglected as well as chain transfer to monomer [43,121,122,123]. All initial oligomeric PS-X is considered to be present in the particles and no exit can occur. For simplicity monomer transport (monomer diffusion between organic and aqueous phase) is ignored. Considering the limited maximum conversion investigated (<95%), the aqueous monomer concentration can be considered constant at its saturated value (4.3 × 10^−3^ mol·L^−1^) [20] and, hence, instantaneous monomer phase transfer to the aqueous phase is assumed. Thermal self-initiation is neglected at the polymerization temperature considered, based on literature data [121,124,125,126].

Diffusional limitations on termination are taken into account through apparent rate coefficients (subscript “app”) that are calculated by means of the composite *k_t_* model [115], also sometimes referred to as the RAFT-chain length dependent-termination (RAFT-CLD-T) model [116]. More information regarding this model and the necessary parameters allowing to account for chain length and monomer conversion dependencies are given in Section S1 of the Supporting Information. The average apparent termination rate coefficient is evaluated using the number radical average radical chain length *x_n,r_*. Note that for termination with chain lengths of 1 the power is 9 in Table 1, as the power of Jonhston-Hall and Monteiro was corrected with a factor of 2, as explained in Derboven et al. [115,116]. As such further improvement of these values is still requested taking into account reported values with other experimental techniques (e.g., power ca. 8) [127]. In the context of the present one should thus realize that termination (at least at low monomer conversions) is fast and hence an establishment of zero-one kinetics is more likely.

As previously indicated already at intermediate monomer conversions RAFT specific reactions, i.e., RAFT addition, fragmentation, cross termination, and transfer (when assuming the degenerative mechanism), can become diffusion controlled, as only macrospecies are involved in similar to conventional termination [48,49,50,52,54,128,129]. Consequently, apparent rate coefficients need to be considered for these RAFT specific reactions as well. A fundamental approach is the coupled parallel encounter pair model, as introduced by D’hooge et al. [50]. In such a model for instance the apparent macro-RAFT addition and fragmentation rate coefficient follow from:

(4) $$k_{add,app\;}={\left(\frac{1}{k_{add,chem}}+\frac{1}{k_{add,diff}}\right)}^{-1}$$

(5) $$k_{frag,app\;}={\left(\frac{1}{k_{frag,chem}}+\frac{K_{eq,1}}{k_{frag,diff}}\right)}^{-1}$$

with k_add/frag,chem_ the intrinsic RAFT macro-addition/fragmentation rate coefficient (see Table 1), k_add/frag,diff_ the diffusional contribution for RAFT macro-addition/fragmentation (which can be approximated by the termination diffusional contribution), and *K_eq,1_* the ratio between the intrinsic macro-RAFT addition and fragmentation rate coefficient, highlighting the coupled nature of these reactions, as even for a unimolecular reaction the two fragments still need to diffuse away from each other as for conventional radical initiation where one has often a cage effect. For a degenerative model the following equations formally result:

(6) $$k_{tr,0,app\;}={\left(\frac{1}{k_{tr,0,chem}}+\frac{1}{k_{tr,0,diff}}+\frac{1}{K_{eq,2}k_{-tr,0,diff}}\right)}^{-1}$$

(7) $$k_{-tr,0,app\;}={\left(\frac{1}{k_{-tr,0,chem}}+\frac{1}{k_{-tr,0,diff}}+\frac{K_{eq,2}}{k_{tr,0,diff}}\right)}^{-1}$$

(8) $$k_{tr,app\;}={\left(\frac{1}{k_{tr,chem}}+\frac{2}{k_{tr,diff}}\right)}^{-1}$$

with *k_(-)tr(,0),app_* the apparent RAFT transfer rate coefficients, *k_(-)tr(,0),chem_* the intrinsic RAFT transfer rate coefficient (see Table 1), *k_(-)tr(,0),diff_* the diffusional contribution for RAFT transfer and *K_eq,2_* equal to:

(9) $$K_{eq,2}=\frac{k_{tr,0,chem}}{k_{-tr,0,chem}}$$

For more information regarding these RAFT apparent rate coefficients, the reader is referred to Section S2 of the Supporting Information. Diffusional limitations on propagation, typically observed at very high monomer conversions (≥95%) [130,131,132] are ignored in the present work, which is justified by investigating the limited maximal monomer conversion. 

### 2.2. Compartmentalization Model: Smith-Ewart and Moment Equations

In RAFT miniemulsion polymerization, some species will be present in each particle in large amounts (a number per particle much higher than 10 for e.g., monomer and *R_*0*_X*) whereas only 0 up to e.g., 10 species of a certain radical type are present in each particle. The concentration of the abundant species can—for simplicity—be approximated by a single average concentration over all particles at any time and if a deterministic solution strategy is followed their concentration vs time evolution is accounted for by means of continuity equations or mass balances [43,50,133,134,135,136]. For the non-abundant species, pseudo-bulk kinetics are very likely not obtained. Here, no averaging is allowed and thus the kinetics can be significantly affected by compartmentalization. This implies that the number of particles with each possible set of a given number of radical types needs to be followed alongside the aforementioned differential equations. So-called multi-dimensional Smith-Ewart equations are therefore needed, with the number of radical types determining the degree of dimensionality [137,138,139,140,141]. 

In our previous work [98] on degenerative RAFT polymerization a two-dimensional Smith-Ewart modeling methodology with the calculation of the number of *R*_0_ and macroradicals per particle has been put forward, accounting for interphase mass transfer and diffusional limitations up to high monomer conversions. Both the calculation of the polymerization rate and average polymer characteristics as a function of time is covered, with the average chain length characteristics following from an extended method of moments. Based on Figure 1—with a non-degenerative model—a five-dimensional Smith-Ewart model is now required and, hence, one can at least theoretically have several radical types be associated with deviations from zero-one kinetics conventionally associated with the propagating macroradical only. The population balances in this model describe the temporal evolution of polymer particles having *k* macroradicals, *l R_0_* radicals, *m R_i_XR*_0_ radicals, *n R_i_XR_j_* radicals and *o R_*0*_XR*_0_ radicals (*N_k,l,m,n,o_*; *k,l,m,n,o* ≥ 0) (Supporting Information: Section S8). The continuity equations of the species in the aqueous phase, the abundant species in the particles and the associated equations for the average chain length characteristics are provided in Supporting Information (Section S9). In these equations, the average numbers of each radical type are defined as:

(10) $$\bar{n}\left(R\right)=\sum_{k,l,m,n,o}k\frac{N_{k,l,m,n,o}}{N_{p}}$$

(11) $$\bar{n}\left(R_{0}\right)=\sum_{k,l,m,n,o}l\frac{N_{k,l,m,n,o}}{N_{p}}$$

(12) $$\bar{n}\left(R_{i}XR_{0}\right)=\sum_{k,l,m,n,o}m\frac{N_{k,l,m,n,o}}{N_{p}}$$

(13) $$\bar{n}\left(R_{i}XR_{j}\right)=\sum_{k,l,m,n,o}n\frac{N_{k,l,m,n,o}}{N_{p}}$$

(14) $$\bar{n}\left(R_{0}XR_{0}\right)=\sum_{k,l,m,n,o}o\frac{N_{k,l,m,n,o}}{N_{p}}$$

in which *N_p_* is the total number of particles.

## 3. Results and Discussion

In this section, attention is first focused on the impact of the RAFT exchange parameters on the main miniemulsion polymerization characteristics, assuming the detailed “non-degenerative” mechanism as defined in Figure 1. Here an average particle size of 100 nm and a targeted chain length (TCL) of 200 ([R_0_X]_0_/[KPS]_0_ = 3, [KPS]_0_ = 4 × 10^−3^ mol·L^−1^, *m_MMA,0_* = 20 g, *m_H2O,0_* = 80 g) are considered for the parameter variations Comb 1–Comb 3 in Table 1. Next a comparison is made with the descriptions following the simplified degenerative mechanism. Finally, it is investigated whether a variation of the average particle size enables the differentiation between the nature of the RAFT mechanism, making a distinction between slow RAFT fragmentation and pronounced RAFT cross-termination. The focus is here on both the changes of the monomer conversion and the polymer properties.

### 3.1. Results Considering a Non-Degenerative Mechanism at an Average Particle Size of 100 nm

**Figure 2:**
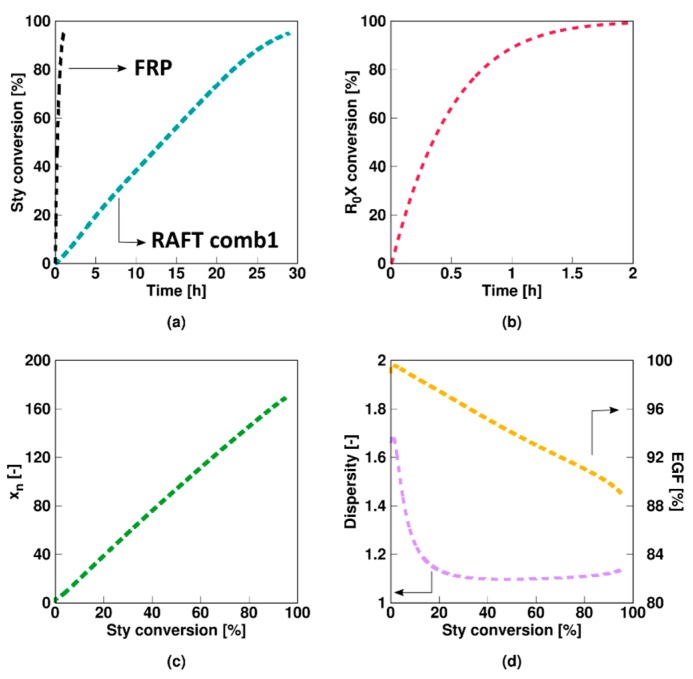
Overview of average characteristics for non-degenerative miniemulsion RAFT polymerization of styrene at 343 K with KPS and an oligomeric RAFT agent. Monomer conversion (**a**) and initial RAFT agent (*R_*0*_X*) conversion (**b**) as a function of time and number average chain length *x_n_* (**c**), dispersity (**d**) and end-group functionality (EGF) (**d**) as a function of styrene conversion. Model parameters for Comb 1 (slow fragmentation case) in Table 1. [Sty]_0_/[R_0_X]_0_ = 200, [R_0_X]_0_/[KPS]_0_ = 3, [KPS]_0_ = 4 × 10^−3^ mol·L^−1^, *m_MMA,0_* = 20 g, *m_H2O,0_* = 80 g; *d_p_* = 100 nm; this slow fragmentation case leads specifically to a much slower polymerization compared to the FRP case (black line in (**a**)).

As shown in Figure 2a, a slow fragmentation model (**Comb 1** in Table 1; non-degenerative parameters) results in a much lower polymerization rate compared to the corresponding FRP. As RAFT intermediate radicals cannot propagate, the low fragmentation rate coefficients involving these species result in a decrease of the macroradical (*R_i_*) amount available for propagation. The low fragmentation rate coefficient of the RAFT intermediate species *R_0_XR*_0_ and *R_i_XR*_0_ results in a prolonged presence of the initial RAFT agent, as shown in Figure 2b. Nevertheless, a linear growth for the number average chain length (*x_n_*; Figure 2c) with monomer conversion and a low dispersity (<1.5, Figure 2d) are obtained. Unavoidable termination events result in a steady decrease of the EGF, although the latter remains high (>88%) as shown in Figure 2d. Overall a reasonable chain growth control is achieved.

**Figure 3:**
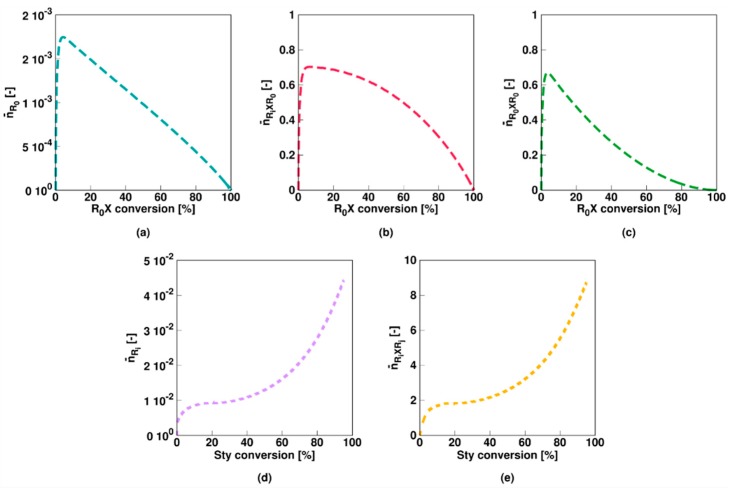
Corresponding evolution of the average number of radicals of a given type (Equation (10)–(14); a–e: RAFT small radical, RAFT intermediate radicals with small RAFT radical, macroradical, and macro-RAFT radical) as a function of initial RAFT agent (*R_*0*_X*) agent or styrene conversion for Figure 2; the slow fragmentation case leads to a strong build-up of the intermediate RAFT radical (see (**e**)) resulting in much higher values then 0.5, the value characteristic value for 0-1 kinetics, from intermediate styrene conversions onwards.

For the slow fragmentation model, the average number of *R*_0_, *R_i_XR*_0_, and *R_*0*_XR*_0_ radicals all steadily decrease as a function of the RAFT agent conversion as shown in Figure 3a–c, due to the formation of macroradicals *R_i_* after addition of monomer to *R*_0_. However, the slow fragmentation results in an initially large (>0.5) average number of *R_i_XR*_0_ and *R_0_XR*_0_ radicals per particle. The average number of *R_i_* radicals is low throughout the entire polymerization (<5 × 10^−2^, Figure 3d) as the radical function is mainly stored in the intermediate *R_i_XR_j_* form (Figure 3e). Above 50% monomer conversion, diffusional limitations on termination (Figure S3 of the Supporting Information) even result in a steep increase of the average number of these radicals (>>2). It can thus be concluded that a multi-dimensional Smith-Ewart description is needed, with a considered maximal radicals per particle beyond a value of 1. In other words, for a slow RAFT fragmentation model zero-one kinetics are not obtained.

**Figure 4:**
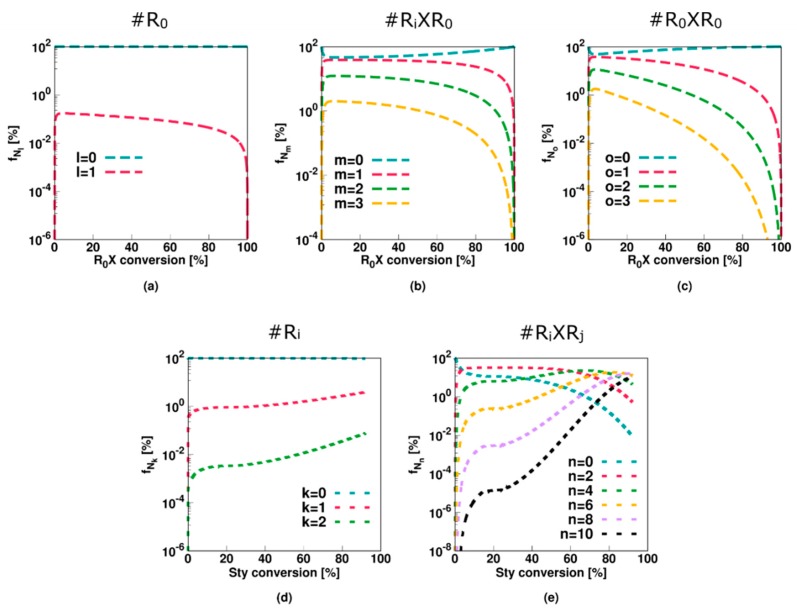
Corresponding fraction of particles with a given number of radicals as a function of the initial RAFT agent (*R_*0*_X*) agent or styrene conversion for Figure 2. Averages are depicted in Figure 3.

This is also confirmed in Figure 4 (Comb 1) displaying the particle distributions. For *R*_0_ and *R_i_* (Figure 4a,d), the high termination, propagation and addition rate coefficients result in almost all particles containing none of these radicals, consistent with the very low average numbers in Figure 3a,d. For *R_i_XR*_0_ and *R_*0*_XR*_0_ radicals (Figure 4a,b), on the other hand, “empty” particles are less present and even at the initial stage of the polymerization process, a 0-1 description is inappropriate. This is even more pronounced for *R_i_XR_j_* radicals as shown in Figure 4e as most particles contain at least one of these radicals already after a few percentages of monomer conversion. At the final stages of the polymerization, a significant amount of particles even contain up to 10 radicals, again highlighting the importance of the use of the multidimensional Smith-Ewart equations. Note that this number is probably high for a true system as the selected parameter combination corresponds to an extreme case of slow RAFT fragmentation.

**Figure 5:**
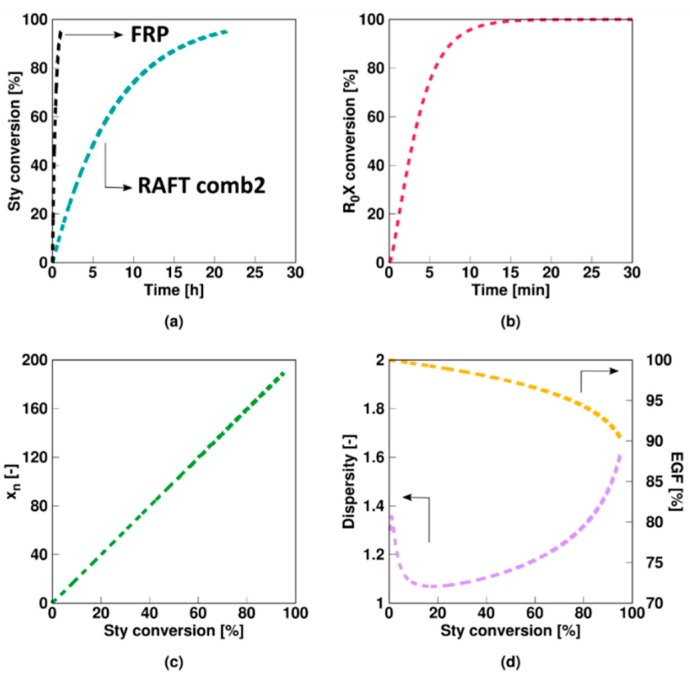
Changes for Figure 2 (Monomer conversion (**a**) and initial RAFT agent (*R_*0*_X*) conversion (**b**) as a function of time and number average chain length *x_n_* (**c**), dispersity (**d**) and end-group functionality (EGF) (**d**) as a function of styrene conversion) in case Comb 2 model parameters from Table 1 instead of Comb 1 model parameters (RAFT cross termination case); a less controlled RAFT polymerization is observed, with again a strongly lower polymerization rate as in the corresponding FRP case.

Figure 5 shows the evolution of the main miniemulsion average characteristics, considering **Comb 2** non-degenerative model parameters of Table 1. Here, in contrast to Comb 1, the intermediate radicals can terminate significantly by reaction with both leaving the group and macroradicals, again resulting in a significant rate reduction compared to the FRP countercase (Figure 5a). The initial RAFT agent is now consumed much faster as shown in Figure 5b. A linear growth of *x_n_* and a low dispersity (Figure 5c,d), except at very high (>85%) monomer conversion again indicate an acceptable controlled character of the polymerization further exemplified by a relatively high EGF (Figure 5d).

**Figure 6:**
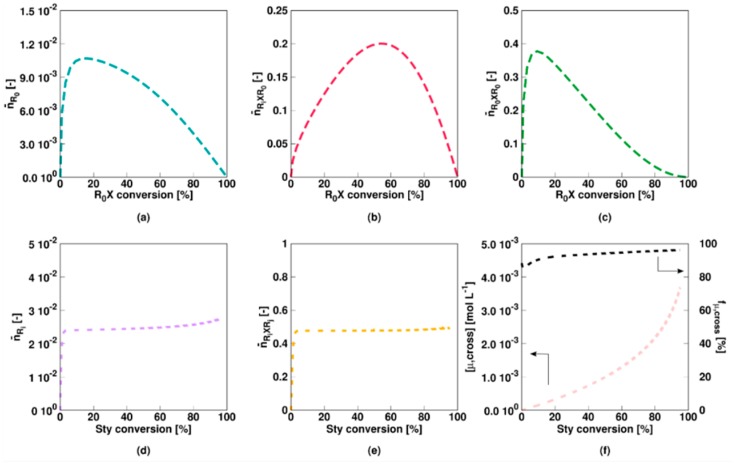
Corresponding evolution of the average number of radicals of a given type as a function of initial RAFT agent (R_0_X) agent or styrene conversion for Figure 5 (Comb 2; **a**–**e**: RAFT small radical, RAFT intermediate radicals with small RAFT radical, macroradical, and macro-RAFT radical), with a value of 0.5 in (**e**). Also subfigure (**f**) with the evolution of the concentration of RAFT-cross termination product (left) and the fraction for this polymer in the overall dead polymer amount (right). Most of the dead polymer is thus star polymer product.

The average number of *R*_0_ radicals per particle for Comb 2 is again very low, as shown in Figure 6a. The average number of *R_i_XR*_0_ and *R_0_XR*_0_ radicals (Figure 6b,c) is slightly lower for Comb 2 as a larger fragmentation rate coefficient is considered and cross-termination is plausible as well. The average number of macroradicals remains low (Figure 6d) and more or less constant, indicating that diffusional limitations do not have a significant effect. Notably the average number of *R_i_XR_j_* radicals (Figure 6e) is constant as well but with a value of roughly 0.5, as cross-termination withholds the build-up of these radical species. As shown in Figure 6f, the concentration of RAFT star cross product steadily increases with increasing monomer conversion and the majority of the dead polymer species is this star product. Hence, for Comb 2, the dominant role of RAFT-cross termination is clear in the radical and dead polymer spectrum.

**Figure 7:**
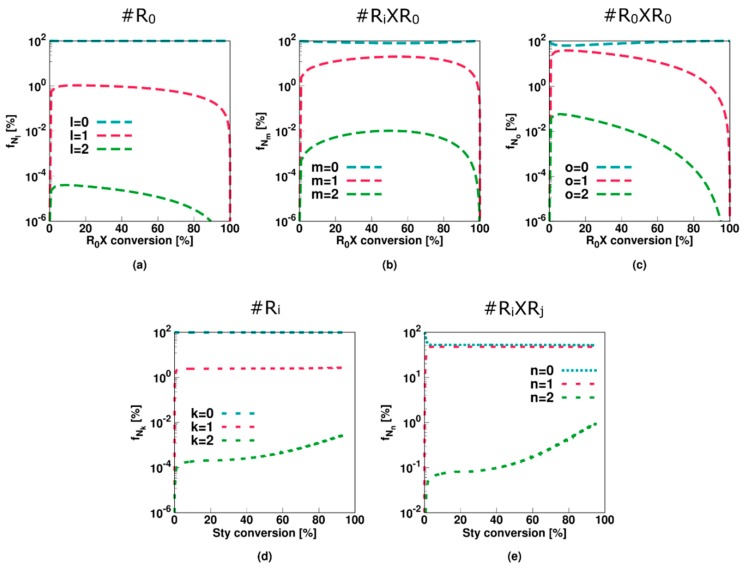
Corresponding fraction of particles with a given number of radicals as a function of initial RAFT agent (*R_*0*_X*) agent or styrene conversion for Figure 5; averages in Figure 6; zero-one kinetics are now established but several radical types are needed.

This conclusion also follows from the radical distributions in Figure 7 with most particles containing no or only a single radical of each radical type. Moreover, for *R_i_XR_j_* radicals (Figure 7e), roughly half the particles contain none of these species whereas the other half contains one, resulting in an average value of approximately 0.5 (Figure 6e). Only at the higher monomer conversions 1% of the particles contain 2 *R_i_XR_j_* radicals, again highlighting the limited impact of diffusional limitations and thus the acceptable approximation of zero-one kinetics, albeit with multiple radical types. 

**Figure 8:**
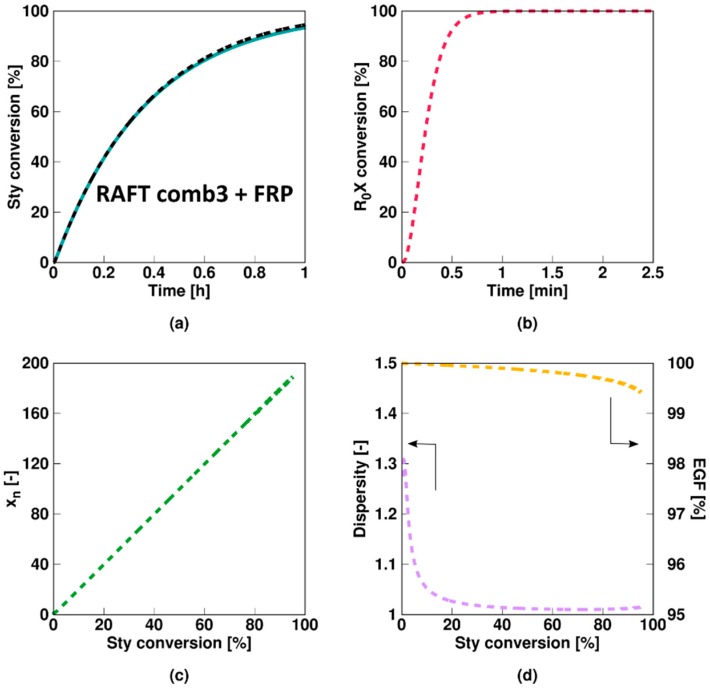
Change of Figure 1 in case Comb 3 model parameters (ideal RAFT agent case) from Table 1 instead of Comb 1 model parameters (Monomer conversion (**a**) and initial RAFT agent (*R_*0*_X*) conversion (**b**) as a function of time and number average chain length *x_n_* (**c**), dispersity (**d**) and end-group functionality (EGF) (**d**) as a function of styrene conversion); identical results are obtained in case a degenerative model is used (see Section S4 in the Supporting Information). This is also consistent with the identical line under the corresponding FRP conditions.

Figure 8 shows the results for **Comb 3**, mimicking an ideal RAFT agent as a high RAFT addition and fragmentation rate coefficient are considered. No rate retardation compared to the FRP case is observed, indicating that the fraction of radicals in the inactive intermediate form is negligible at any time and that the gel-effect impact as for the bulk case is much less an issue, also bearing in mind that the monomer is styrene, which is less prone to rate acceleration as e.g., methyl methacrylate polymerization [48]. Moreover, the RAFT agent is almost instantaneously consumed completely, as shown in Figure 8b. It can be confirmed that a RAFT agent characterized by parameter set Comb 3 behaves as an ideal RAFT agent as *x_n_* increases linearly with monomer conversion, the dispersity is very close to the theoretical lower limit of 1, and the EGF is >99% (Figure 8b–d). 

**Figure 9:**
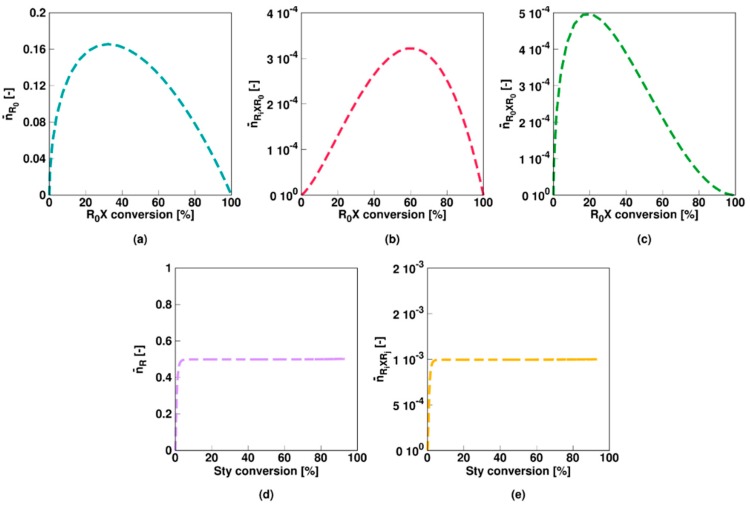
Corresponding evolution of the average number of radicals of a given type as a function of initial RAFT agent (R_0_X) agent or styrene conversion for Figure 9; **a**–**e**: RAFT small radical, RAFT intermediate radicals with small RAFT radical, macroradical, and macro-RAFT radical; for (**d**) an average value of 0.5, highlighting the targeted instantaneous termination with conventional macroradicals and the validity of zero-one kinetics.

As mentioned previously, a RAFT polymerization and corresponding FRP conversion profile can only coincide if the RAFT intermediate radicals are formed rapidly and are extremely short-lived, which is confirmed by the average radical values for Comb 3 shown in Figure 9. A constant value of 0.5 is obtained for the average number of macroradicals (Figure 9d), indicating instantaneous termination if two macroradicals are present in a particle. Under the Comb 3 miniemulsion conditions the assumption of zero-one kinetics is thus again acceptable.

**Figure 10:**
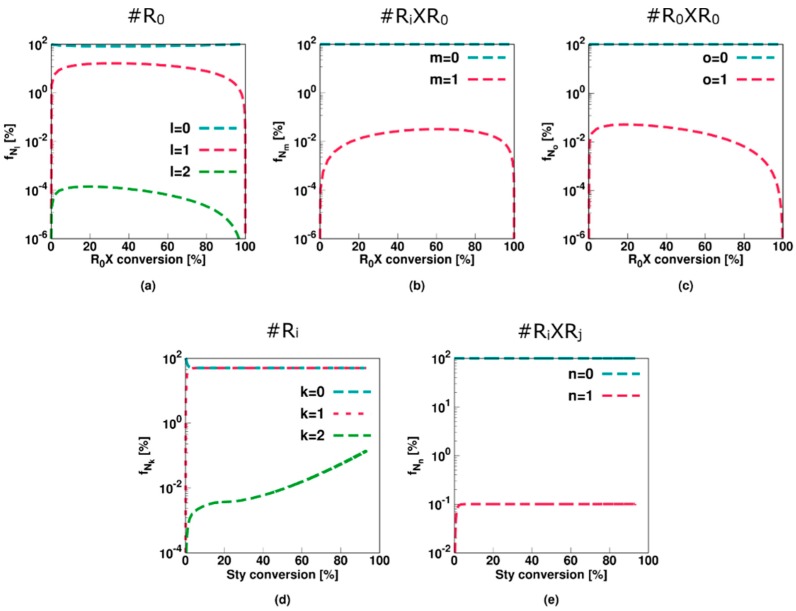
Corresponding fraction of particles with a given number of radicals as a function of initial RAFT agent (*R_*0*_X*) agent or styrene conversion for Figure 9; averages in Figure 10.

The average values of Figure 9 (Comb 3) can be further understood by considering the particle type distributions of Figure 10. The only radical type that can be significantly present in a particle is a macroradical. More specifically, half of the particles contain zero macroradicals whereas the other half contain only one macroradical. As opposed to Comb 2 the key 0.5 value is thus now established with the population of macroradicals.

### 3.2. Relevance of Description with an Explicit Calculation of the RAFT Intermediate

**Figure 11:**
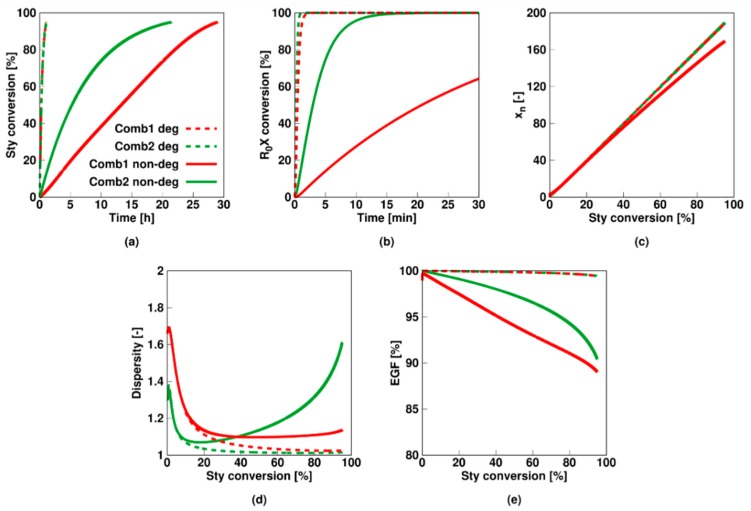
Comparison of non-degenerative results (full lines) of Figure 2/Figure 5 with the simplified degenerative model results (dashed lines) for Comb 1 (red)/Comb 2 (green) model parameters in Table 1; monomer conversion (**a**) and initial RAFT agent (*R_*0*_X*) conversion (**b**) as a function of time and number average chain length *x_n_* (**c**), dispersity (**d**) and end-group functionality (EGF) (**e**) as a function of styrene conversion; The non-degenerative models are needed for these combinations. For Comb 3, the degenerative description is allowed (see Supporting Information).

The next logical step is to explicitly validate whether a simplified degenerative mechanism (no explicit calculation concentration RAFT intermediate in models) obtains the detailed kinetic descriptions as produced with the non-degenerative mechanism (explicit calculation concentration of RAFT intermediate). As expected for Comb 3 (ideal RAFT agent) this is case (Figure S2 in the Supporting Information). For the other two combinations this is not the case as shown in Figure 11 (dashed lines: non-degenerative description from before; full lines: degenerative approximation; Comb 1: red; Comb 2: green). It follows that for Comb 1 (red lines) both the styrene (Figure 11a) and RAFT agent consumption rate (Figure 11b) are significantly reduced when the intermediate radicals are considered compared to the simplified degenerative approximation. This is not surprising as the slow fragmentation results in the formation of radicals incapable of propagating whereas in the degenerative approach the RAFT mechanism is described by a direct exchange which does formally not include the formation of these intermediate species. The *x_n_* (Figure 11c) and dispersity (Figure 11d) are only minorly affected by switching from the non-degenerative to the degenerative approach. The EGF is however significantly lower for the non-degenerative description but this is related to the longer polymerization time needed to reach the final conversion. 

Similarly, for Comb 2 (green lines), the monomer and initial RAFT agent conversion are again much slower for the non-degenerative description as not only the non-propagating intermediate species are explicitly taken into account but these can also terminate with macroradicals. Nonetheless, the *x_n_* is unaffected as both models are coincident. However, in the non-degenerative approach, the domination event is RAFT cross-termination, resulting in star product whereas in the degenerative approach only conventional termination can occur, resulting in linear dead species. As the star product will inherently be larger than the linear dead product, the overall dispersity for the non-degenerative description will be larger as well, which can be seen in Figure 11d. Finally, the EGF for the non-degenerative description is again much lower due to the prolonged reaction time. Hence, only for very well-defined RAFT systems the degenerative assumption is afforded.

### 3.3. Relevance of the (Average) Particle Size in View of Future Experimental Analysis

**Figure 12:**
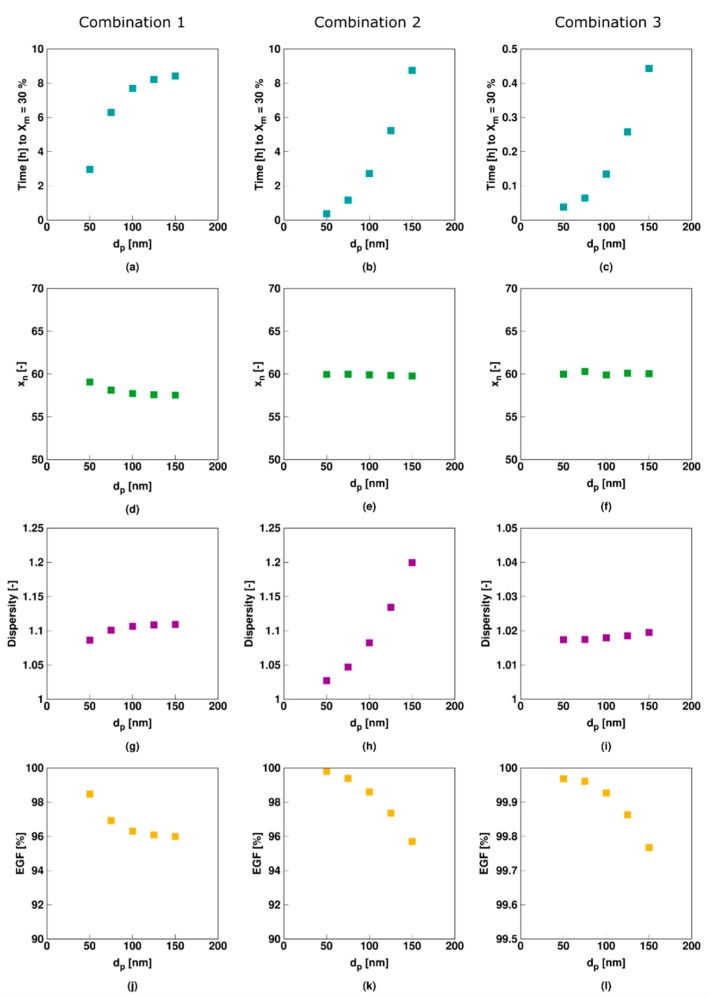
Influence of the (average) particle size (*d_p_*) on the time to reach 30% monomer conversion *X_M_* ((**a**)–(**c**), blue), the number average chain length (*x_n_*, (**d**)–(**f**), green), dispersity ((**g**)–(**i**), purple) and EGF ((**j**)–(**l**), orange) for model parameters Comb 1 (first column), Comb 2 (second column), and Comb 3 (third column) in Table 1. [Sty]_0_/[R_0_X]_0_ = 200, [R_0_X]_0_/[KPS]_0_ = 3, [KPS]_0_ = 4 × 10^−3^ mol·L^−1^, *m_MMA,0_* = 20 g, and *m_H2O,0_* = 80 g. *X_M_* is 30% and *X_R0X_* is 100% for all simulation points.

Figure 12 shows the influence of *d_p_* on the reaction time to reach a monomer conversion (*X_M_*) of 30% and the main average polymer properties at this *X_M_* for the three model parameter combinations in Table 1, with Column *i* relating to Comb *i* and *d_p_* varying between 50 and 150 nm. This low monomer conversion is selected for illustration purposes and to avoid too long simulation times. It is clear that for all three models the time to reach a *X_M_* of 30% increases with increasing *d_p_* (Figure 12a–c. Nevertheless, for Comb 1 (Figure 12a) the increase slows down with increasing *d_p_* whereas for Comb 2 (Figure 12b) and Comb 3 (Figure 12c) the increases become more pronounced. The EGF decreases (Figure 12j–l) are related to the monomer conversion changes, with higher losses implying more conventional radical initiator consumption before the targeted monomer conversion is reached. This again illustrates the link between the reaction time and the loss of the RAFT polymerization livingness and further highlights the relevance of EGF measurements. 

In contrast, regardless of the parameter combination, *x_n_* is practically unaffected by *d_p_* (Figure 12d–f), which is consistent with the general observation under bulk/solution conditions that this average polymer response is less sensitive to process variations. The relation between *d_p_* and dispersity (Figure 12g–i) is strongly varied depending on the parameters combination, highlighting the potential of dispersity experimental analysis besides conventional monomer conversion measurements to study the nature of possible RAFT retardations. For Comb 1 a plateau behavior results with increasing *d_p_* with dispersity values around 1.1, whereas for Comb 2 an accelerated increase is obtained with increasing *d_p_* with values above 1.2. For Comb 3, on the other hand, a plateau behavior with decreasing *d_p_* is observed, with although always very low dispersity values (close to 1). 

**Figure 13:**
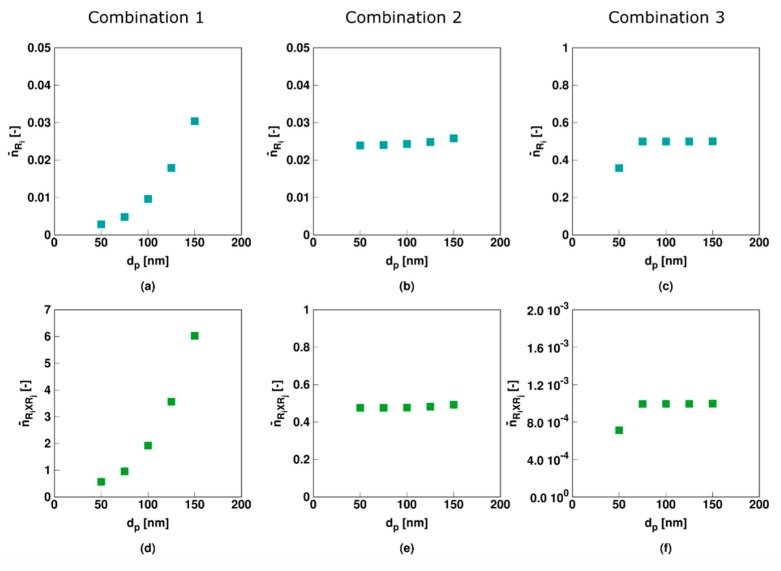
Corresponding average number of macro-(top) and RAFT intermediate radicals (bottom) for Figure 12 (*X_M_* of 30%; Comb 1–3; left to middle column); for completeness other average radical numbers in Figure S4 of the Supporting Information.

As shown in Figure 13, only for Comb 1 (slow RAFT fragmentation) the average number of macroradicals and *R_i_XR_j_* radicals per particle is significantly affected by the average particle size. Besides for Comb 3 at a small particle size of 50 nm, both Comb 2 and Comb 3 are rather unaffected, with notably average values around 0.5 for the RAFT intermediate radical in Comb 2 and the conventional macroradical in Comb 3. This implies the establishment of a kind of balancing of exchange reaction rates in Comb 2 and Comb 3 which is not met in Comb 1 where the intermediate RAFT radical cannot easily disappear, as there is slow RAFT fragmentation and no RAFT cross-termination. 

**Figure 14:**
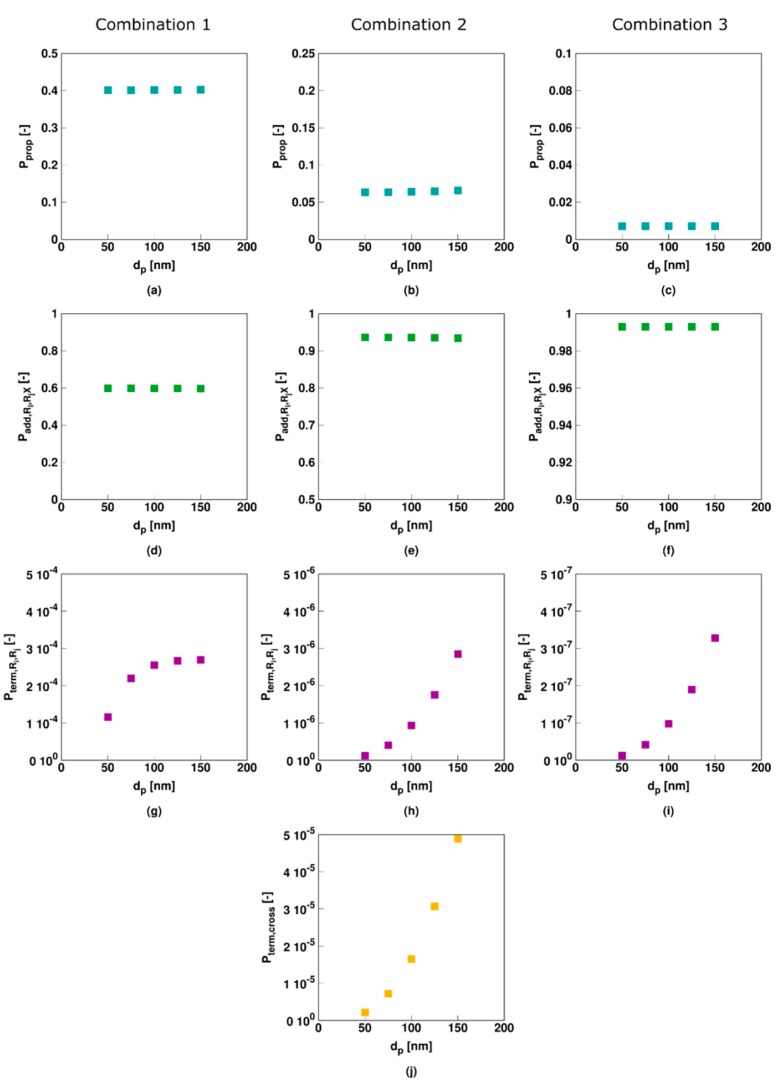
Corresponding radical reaction probabilities for the macroradicals for Figure 12 (*X_M_* of 30%); top to bottom: propagation, RAFT addition, (conventional) termination and RAFT cross-termination; columns: left to right: Comb 1 to Comb 3; For completeness, RAFT-intermediate radical rates in Figure S5 of the Supporting Information.

This can be further investigated through the radical reaction probabilities shown in Figure 14 that are calculated based on all particles jointly. It follows that the probabilities of propagation and addition (first two rows) are unaffected by *d_p_* for all three parameter combinations as these reactions involve, respectively, monomer and dormant species which are present in relatively large concentrations in each particle so that compartmentalization does not matter, at least in a Smith–Ewart based modeling approach. In contrast (RAFT cross-) termination involves radicals only and as a result when the related probabilities (last two rows) vary with particle size. Similar to the polymerization rate trends (related to Figure 12a–c), these termination probabilities (Figure 14g–j) increase with increasing *d_p_* with the increase slowing down for Comb 1 and increasing for Comb 2 and Comb 3. For Comb 3 the termination probability is as such extremely low (e.g., 10^−7^) and its actual impact is almost negligible, highlighting again the ideal nature of this RAFT agent. 

Hence, the relation between the parameter combination for RAFT exchange and the *d_p_* effect in Figure 12 needs to be related to the relative importance of termination, in agreement with the suggestions by Tobita et al. [92,93,94] For Comb 2 the dominant termination rate is the RAFT-cross termination rate (probabilities of e.g., 10^−5^), with the conventional radical termination rate matching the *d_p_* dependence of RAFT-cross termination but a negligible effect as such due to too low probabilities (e.g., 10^−6^). A lower *d_p_* leads in Comb 2 to more segregation, stating that two species can only react when they are present in the same particle, blocking more cross-termination, which results in a stronger polymerization rate acceleration until a *d_p_* is reached at which cross-termination becomes kinetically insignificant. An acceleration is also obtained with Comb 1, as there the segregation effect on the conventional termination is still active with low but still significant probabilities (e.g., 10^−4^). Here the *d_p_* dependence is although different with at the higher *d_p_* – in the studied *d_p_* range- bulk-like behavior and thus constant reaction probabilities. Such different dependence is also reflected in the evolutions for the average polymer properties, increasing the potential to explain rate retardations in RAFT miniemulsion polymerization. It is clear that the identification of a rate acceleration is insufficient as both a slow RAFT transfer and a polymerization with significant RAFT cross-termination are characterized by such feature. Only upon a close inspection of the *d*_p_ dependency and other characteristics is a proper distinction theoretically possible.

## 5. Conclusions

The added value of studying miniemulsion RAFT polymerization in a range of synthesis conditions to clarify the nature of a possible RAFT retardation is theoretically highlighted based on multi-dimensional Smith–Ewart equations. Essential is the effect of the average particle size on the relative importance of termination reactions in view measureable characteristics. In future experimental work, focus should be not restricted to examining differences in the monomer conversion profile for slow RAFT fragmentation and significant cross-termination but also the relation between the average particle size and the control over average properties should be investigated. In particular, the measured dispersity and end-group functionality variation with *d_p_* can be used to differentiate slow RAFT fragmentation from significant RAFT-cross termination. Moreover, a comparison with the FRP conversion profile is still recommended to confirm whether the RAFT agent can be considered as ideal.

The developed modeling tool can be used to better understand RAFT miniemulsion kinetics. For a slow RAFT fragmentation, zero-one kinetics cannot be assumed for the related radical type, at least if the particle size is sufficiently high. For a fast RAFT cross-termination zero-one kinetics are obtained but multiple radical types need to be considered and the dead polymer product is dominantly star product, which opens the pathway to further experimental validation. Once the degenerative mechanism can be safely assumed, which in a modeling context implies that the RAFT intermediate concentration does not need to be calculated explicitly, a direct switch can be made from a five- to a two-dimensional Smith-Ewart equation based description, strongly simplifying the overall kinetic description and design.

## Figures and Tables

**Table 1:** Overview of reactions and intrinsic rate coefficients for RAFT miniemulsion polymerization of styrene at 343 K, with initiator related coefficients based on KPS and a differentiation between RAFT exchange parameters: Combination 1–3 (slow RAFT fragmentation/high RAFT cross-termination/ideal RAFT agent). Further differentiation between reactions for a simplified degenerative model (DM) and non-degenerative model (NDM) as defined in Figure 1; an oligomeric styrene RAFT agent is assumed; all rate coefficients in L mol^−1^·s^−1^ except *k_dis_* and *k_frag,R0/I,R0/IX_*; also included interphase mass transfer parameters; for termination and RAFT exchange apparent rate coefficients are used (Section S1 and S2 of the Supporting Information); ∨: considered.

Reaction	DM	NDM	Equation	*Comb 1*	*Comb 2*	*Comb 3*	*Ref*
***Aqueous phase reactions***							
**Diss.^(a),(b)^ of *I*_2_**	∨	∨	$I_{2,aq}\overset{f,k_{dis}}{\to }2I_{aq}^{•}$	$2.2\times 10^{-5}$			[105]
**Chain ini *I***	∨	∨	$I_{aq}^{•}+M_{aq}\overset{k_{pI}}{\to }R_{1,aq}^{•}$	$4.9\times \;10^{5}$			[113]
**Propagation ^(c),(d)^**	∨	∨	$R_{1,aq}^{•}+M_{aq}\overset{k_{p}}{\to }R_{2,aq}^{•}$	$4.8\times 10^{2}$			[114]
***Organic phase reactions***							
$\mathrm{Chain}\;\mathrm{ini}\;R_{0}^{•}$ **^(e)^**	∨	∨	$R_{0,p}^{•}+M_{p}\overset{k_{pR_{0}}}{\to }R_{1,p}^{•}$	$4.8\times 10^{2}$			[114]
**Propagation**	∨	∨	$R_{i,p}^{•}+M_{p}\overset{k_{p}}{\to }R_{i+1,p}^{•}$	$4.8\;\times 10^{2}$			[114]
**Termination ^(f)^**	∨	∨	$R_{0,p}^{•}+R_{0,p}^{•}\overset{k_{tc,00}}{\to }P_{0,p}$	$1.0\;\times 10^{9}$			[115,116]
**^(f)^**	∨	∨	$R_{0,p}^{•}+R_{i,p}^{•}\overset{k_{tc,0}}{\to }P_{i,p}$	$1.0\;\times 10^{9}$			[115,116]
**^(f)^**	∨	∨	$R_{i,p}^{•}+R_{j,p}^{•}\overset{k_{tc}^{i,j}}{\to }P_{i+j,p}$	$1.0\;\times 10^{9}$			[115,116]
**RAFT add/frag ^(i)^**		∨	$R_{0,p}+{\left(R_{i}X\right)}_{p}\overset{k_{add,R_{0},R_{i}X}}{\to }{\left(R_{0}XR_{i}\right)}_{p}$	$1.0\;\times 10^{5}$	$1.0\times 10^{6}$	$1.0\;\times 10^{7}$	^(j)^
**^(i)^**		∨	$R_{i,p}+{\left(R_{j}X\right)}_{p}\overset{k_{add,R_{i},R_{j}X}}{\to }{\left(R_{i}XR_{j}\right)}_{p}$	$1.0\;\times 10^{5}$	$1.0\times 10^{6}$	$1.0\times 10^{7}$	^(j)^
**^(i)^**		∨	$R_{0,p}+{\left(R_{0}X\right)}_{p}\overset{k_{add,R_{0},R_{0}X}}{\to }{\left(R_{0}XR_{0}\right)}_{p}$	$1.0\times 10^{5}$	$1.0\;\times 10^{6}$	$1.0\;\times 10^{7}$	^(j)^
**^(i)^**		∨	$R_{i,p}+{\left(R_{0}X\right)}_{p}\overset{k_{add,R_{i},R_{0}X}}{\to }{\left(R_{i}XR_{0}\right)}_{p}$	$1.0\;\times 10^{5}$	$1.0\;\times 10^{6}$	$1.0\;\times 10^{7}$	^(j)^
**^(a),(i)^**		∨	${\left(R_{0}XR_{i}\right)}_{p}\overset{k_{frag,R_{0},R_{i}X}}{\to }R_{0,p}+{\left(R_{i}X\right)}_{p}$	$1.0\;\times 10^{1}$	$1.0\;\times 10^{3}$	$1.0\;\times 10^{8}$	^(j)^
**^(a),(i)^**		∨	${\left(R_{0}XR_{i}\right)}_{p}\overset{k_{frag,R_{i},R_{0}X}}{\to }R_{i,p}+{\left(R_{0}X\right)}_{p}$	$1.0\times 10^{1}$	$1.0\times 10^{3}$	$1.0\;\times 10^{8}$	^(j)^
**^(a),(i)^**		∨	${\left(R_{i}XR_{j}\right)}_{p}\overset{k_{frag,R_{i},R_{j}X}}{\to }R_{i,p}+{\left(R_{j}X\right)}_{p}$	$1.0\;\times 10^{1}$	$1.0\;\times 10^{3}$	$1.0\;\times 10^{8}$	^(j)^
**^(a),(i)^**		∨	${\left(R_{0}XR_{0}\right)}_{p}\overset{k_{frag,R_{0},R_{0}X}}{\to }R_{0,p}+{\left(R_{0}X\right)}_{p}$	$1.0\;\times 10^{1}$	$1.0\;\times 10^{3}$	$1.0\;\times 10^{8}$	^(j)^
**RAFT cross-t^(i)^**		∨	$R_{i,p}+{\left(R_{j}XR_{0}\right)}_{p}\overset{k_{tcross}}{\to }P_{i+j,p}$	0	$1.0\times 10^{8}$	0 ^(k)^	^(j)^
**^(i)^**		∨	$R_{i,p}+{\left(R_{j}XR_{k}\right)}_{p}\overset{k_{tcross}}{\to }P_{i+j+k,p}$	0	$1.0\times 10^{8}$	0	^(j)^
**^(i)^**		∨	$R_{i,p}+{\left(R_{0}XR_{0}\right)}_{p}\overset{k_{tcross}}{\to }P_{i,p}$	0	$1.0\times 10^{8}$	0	^(j)^
**^(i)^**		∨	$R_{0,p}+{\left(R_{i}XR_{0}\right)}_{p}\overset{k_{tcross}}{\to }P_{i,p}$	0	$1.0\times 10^{8}$	0	^(j)^
**^(i)^**		∨	$R_{0,p}+{\left(R_{i}XR_{j}\right)}_{p}\overset{k_{tcross}}{\to }P_{i+j,p}$	0	$1.0\times 10^{8}$	0	^(j)^
**^(i)^**		∨	$R_{0,p}+{\left(R_{0}XR_{0}\right)}_{p}\overset{k_{tcross}}{\to }P_{0,p}$	0	$1.0\times 10^{8}$	0	^(j)^
**RAFT exchange**	∨		$R_{i,p}^{•}+R_{0}X_{p}\overset{k_{tr,0}}{\to }R_{i}X_{p}+R_{0,p}^{•}$	$5.0\times 10^{4}$	$5.0\times 10^{5}$	$5.0\times 10^{6}$	^(g)^
	∨		$R_{0,p}^{•}+R_{i}X_{p}\overset{k_{-tr,0}}{\to }R_{0}X_{p}+R_{i,p}^{•}$	$5.0\times 10^{4}$	$5.0\times 10^{5}$	$5.0\;\times 10^{6}$	^(g)^
	∨		$R_{i,p}^{•}+R_{j}X_{p}\overset{k_{tr}}{\to }R_{i}X_{p}+R_{j,p}^{•}$	$5.0\times 10^{4}$	$5.0\times 10^{5}$	$5.0\times 10^{6}$	^(g)^
***interphase mass transport***							
**Entry of** $R_{2,aq}^{•}$	∨	∨	$R_{2,aq}^{•}\overset{k_{entryR_{2}}}{\to }R_{2,p}^{•}$	$5.0\;\times 10^{7}$			^(d),(h)^

(a) s^−1^; (b) *f* = 0.2 (middle of literature [117] range of 0.1–0.3); (c) considered equal to *k_p_* to a first approximation; (d) critical chain length for the entry of styrene is 2; (e) equal to *k_p_* due to oligomeric RAFT agent; (f) given value is that of $k_{t,app}^{1,1}$, see Section S1 in the Supporting Information for monomer and chain length dependencies; only with termination by recombination; correction with factor 2 and based on previous work [115,116]. (g) calculated from corresponding addition and fragmentation rate coefficients (Equation (6)–(8); (h) large values are assumed to reflect the strong entry rate of molecules possessing the critical chain length and values given for *d_p_* = 1.0 × 10² nm; (i) given value is the intrinsic rate coefficient, for calculation of the apparent rate coefficients see Section S2 in the supporting information; (j) this work; (k) can be taken as 0 as kinetically insignificant (Figure S1 of the Supporting Information).

## Supplementary Materials

The supplementary materials are available online at http://www.mdpi.com/2073-4360/11/2/320/s1.

## References

[1] Thickett S.C., Gilbert R.G. (2007). Emulsion polymerization: State of the art in kinetics and mechanisms. Polymer (Guildf.).

[2] Cunningham M.F. (2008). Controlled/living radical polymerization in aqueous dispersed systems. Prog. Polym. Sci..

[3] Destarac M. (2010). Controlled radical polymerization: Industrial stakes, obstacles and achievements. Macromol. React. Eng..

[4] Zetterlund P.B., Thickett S.C., Perrier S., Bourgeat-Lami E., Lansalot M. (2015). Controlled/Living Radical Polymerization in Dispersed Systems: An Update. Chem. Rev..

[5] Matyjaszewski K., Spanswick J. (2005). Controlled/living radical polymerization. Materialstoday.

[6] Grishin D.F., Grishin I.D. (2011). Controlled radical polymerization: Prospects for application for industrial synthesis of polymers (Review). Russ. J. Appl. Chem..

[7] Zetterlund P.B., Kagawa Y., Okubo M. (2008). Controlled/living radical polymerization in dispersed systems. Chem. Rev..

[8] Matyjaszewski K. (2012). Controlled radical polymerization: State-of-the-art in 2011. ACS Symp. Ser..

[9] Save M., Guillaneuf Y., Gilbert R.G. (2006). Controlled radical polymerization in aqueous dispersed media. Aust. J. Chem..

[10] Asua J.M. (2007). Polymer Reaction Engineering.

[11] Matyjaszewski K. (1996). Controlled Radical Polymerization: Mechanisms. Curr. Opin. Solid State Mater. Sci..

[12] Matyjaszewski K. (2003). Advances in Controlled/Living Radical Polymerization.

[13] Barner L., Davis T.P., Stenzel M.H., Barner-Kowollik C. (2007). Complex macromolecular architectures by reversible addition fragmentation chain transfer chemistry: Theory and practice. Macromol. Rapid Commun..

[14] Fierens S., D’hooge D., Van Steenberge P., Reyniers M.-F., Marin G. (2015). Exploring the Full Potential of Reversible Deactivation Radical Polymerization Using Pareto-Optimal Fronts. Polymers (Basel).

[15] D’Hooge D.R., Van Steenberge P.H.M., Reyniers M.F., Marin G.B. (2014). Fed-batch control and visualization of monomer sequences of individual ICAR ATRP gradient copolymer chains. Polymers (Basel).

[16] Brandl F., Drache M., Beuermann S. (2018). Kinetic Monte Carlo Simulation Based Detailed Understanding of the Transfer Processes in Semi-Batch Iodine Transfer Emulsion Polymerizations of Vinylidene Fluoride. Polymers (Basel).

[17] Barner-Kowollik C. (2008). Handbook of RAFT Polymerization.

[18] Moad G., Chiefari J., Chong Y.K., Krstina J., Mayadunne R.T.A., Postma A., Rizzardo E., Thang S.H. (2000). Living free radical polymerization with reversible addition–fragmentation chain transfer (the life of RAFT). Polym. Int..

[19] De Rybel N., Van Steenberge P.H.M., Reyniers M.-F., D’hooge D.R., Marin G.B. (2018). How chain length dependencies interfere with the bulk RAFT polymerization rate and microstructural control. Chem. Eng. Sci..

[20] Gilbert R.G. (1995). Emulsion Polymerization: A Mechanistic Approach.

[21] Charleux B., Monteiro M.J., Heuts H. (2013). Living Radical Polymerisation in Emulsion and Miniemulsion. Chemistry and Technology of Emulsion Polymerisation.

[22] Rawlston J.A. (2010). Multiscale Modeling of Free-Radical Polymerization Kinetics. Ph.D. Thesis.

[23] Prescott S.W., Ballard M.J., Rizzardo E., Gilbert R.G. (2006). Rate optimization in controlled radical emulsion polymerization using RAFT. Macromol. Theory Simul..

[24] Asua J.M. (2014). Challenges for industrialization of miniemulsion polymerization. Prog. Polym. Sci..

[25] Matyjaszewski K., Davies T.P. (2002). Handbook of Radical Polymerisation.

[26] Mastan E., Li X., Zhu S. (2015). Modeling and theoretical development in controlled radical polymerization. Prog. Polym. Sci..

[27] D’hooge D.R., Van Steenberge P.H.M., Reyniers M.-F., Marin G.B. (2016). The strength of multi-scale modeling to unveil the complexity of radical polymerization. Prog. Polym. Sci..

[28] Zetterlund P.B. (2011). Controlled/living radical polymerization in nanoreactors: Compartmentalization effects. Polym. Chem..

[29] Zetterlund P.B., Okubo M. (2006). Compartmentalization in nitroxide-mediated radical polymerization in dispersed systems. Macromolecules.

[30] Cano-Valdez A., Saldívar-Guerra E., González-Blanco R., Cunningham M.F., Herrera-Ordóñez J. (2017). Nitroxide Mediated Radical Emulsion Polymerization: Mathematical Modeling. Macromol. Symp..

[31] Tobita H. (2011). Threshold particle diameters in miniemulsion reversible-deactivation radical polymerization. Polymers (Basel).

[32] Prescott S.W., Ballard M.J., Rizzardo E., Gilbert R.G. (2005). Radical Loss in RAFT-mediated emulsion polymerizations. Macromolecules.

[33] Liu S., Hermanson K.D., Kaler E.W. (2006). Reversible Addition−Fragmentation Chain Transfer Polymerization in Microemulsion. Macromolecules.

[34] Qiu J., Charleux B., Matyjaszewski K. (2001). Controlled/living radical polymerization in aqueous media: Homogeneous and heterogeneous systems. Prog. Polym. Sci..

[35] Monteiro M.J., Cunningham M.F. (2012). Polymer Nanoparticles via Living Radical Polymerization in Aqueous Dispersions: Design and Applications. Macromolecules.

[36] Monteiro M.J., Hodgson M., De Brouwer H. (2000). Influence of RAFT on the rates and molecular weight distributions of styrene in seeded emulsion polymerizations. J. Polym. Sci. Part A Polym. Chem..

[37] Landfester K., Willert M., Antonietti M. (2000). Preparation of polymer particles in nonaqueous direct and inverse miniemulsions. Macromolecules.

[38] Ting S.R.S., Min E.H., Zetterlund P.B. (2011). Reversible AdditionFragmentation Chain Transfer (RAFT) polymerization in miniemulsion based on in situ surfactant generation. Aust. J. Chem..

[39] Jansen T.G.T., Meuldijk J., Lovell P.A., van Herk A.M. (2015). On the Reaction Characteristics of Miniemulsion Polymerization with Aqueous Phase Initiation - Experiments and Modeling. Macromol. React. Eng..

[40] Perrier S., Takolpuckdee P. (2005). Macromolecular design via reversible addition-fragmentation chain transfer (RAFT)/xanthates (MADIX) polymerization. J. Polym. Sci. Part A Polym. Chem..

[41] Yang L., Luo Y., Li B. (2006). The Influence of Surfactant Coverage of the Minidroplets on RAFT Miniemulsion Polymerization. J. Polym. Sci. Part A Polym. Chem..

[42] Derboven P., Van Steenberge P.H.M., Reyniers M., Barner-kowollik C., Dagmar R.D., Marin G.B., D’hooge D.R., Marin G.B., Dagmar R.D., Marin G.B. (2016). Chain transfer in degenerative RAFT polymerization revisited: A comparative study. Macromol. Theory Simul..

[43] Devlaminck D.J.G., Van Steenberge P.H.M., De Keer L., Reyniers M.-F., D’hooge D.R. (2017). A detailed mechanistic study of bulk MADIX of styrene and its chain extension. Polym. Chem..

[44] Kubo K., Goto A., Sato K., Kwak Y., Fukuda T. (2005). Kinetic study on reversible addition-fragmentation chain transfer (RAFT) process for block and random copolymerizations of styrene and methyl methacrylate. Polymer (Guildf.).

[45] Moad G., Flagship C.M., Ave B. (2015). Controlled Radical Polymerization: Mechanisms.

[46] Haven J.J., Junkers T. (2018). Mapping dithiobenzoate-mediated RAFT polymerization products via online microreactor/mass spectrometry monitoring. Polymers (Basel).

[47] Derboven P., Van Steenberge P., Reyniers M.-F., Barner-Kowollik C., D’hooge D.R., Marin G.B. (2016). A novel method for the measurement of degenerative chain transfer coefficients: Proof of concept and experimental validation. Polym. Chem..

[48] De Rybel N., Van Steenberge P.H.M., Reyniers M.-F., Barner-Kowollik C., D’hooge D.R., Marin G.B. (2017). An Update on the Pivotal Role of Kinetic Modeling for the Mechanistic Understanding and Design of Bulk and Solution RAFT Polymerization. Macromol. Theory Simul..

[49] Wang A.R., Zhu S. (2003). Effects of Diffusion-Controlled Radical Reactions on RAFT Polymerization. Macromol. Theory Simul..

[50] D’hooge D.R., Reyniers M.F., Marin G.B. (2013). The crucial role of diffusional limitations in controlled radical polymerization. Macromol. React. Eng..

[51] Devlaminck D.J.G., Van Steenberge P.H.M.M., Reyniers M.-F.M.-F., D’hooge D.R.D.R. (2018). Deterministic modeling of RAFT miniemulsion conversion and average chain length characteristics: Invalidity of zero-one nature at higher monomer conversions. Macromolecules.

[52] Peklak A.D., Butté A. (2006). Modeling of diffusion limitations in bulk RAFT polymerization. Macromol. Theory Simul..

[53] Achilias D.S. (2007). A review of modeling of diffusion controlled polymerization reactions. Macromol. Theory Simul..

[54] Barner-Kowollik C., Russell G.T. (2009). Chain-length-dependent termination in radical polymerization: Subtle revolution in tackling a long-standing challenge. Prog. Polym. Sci..

[55] O’Driscoll K.F. (1989). Comprehensive Polymer Science.

[56] Russell G.T. (2002). The kinetics of free-radical polymerization: Fundamental aspects. Aust. J. Chem..

[57] McLeary J.B., Klumperman B. (2006). RAFT mediated polymerisation in heterogeneous media. Soft Matter.

[58] Barner-Kowollik C., Quinn J.F., Morsley D.R., Davis T.P. (2001). Modeling the reversible addition-fragmentation chain transfer process in cumyl dithiobenzoate-mediated styrene homopolymerizations: Assessing rate coefficients for the addition-fragmentation equilibrium. J. Polym. Sci. Part A Polym. Chem..

[59] Monteiro M.J., De Brouwer H. (2001). Intermediate radical termination as the mechanism for retardation in reversible addition-fragmentation chain transfer polymerization. Macromolecules.

[60] Moad G. (2014). Mechanism and Kinetics of Dithiobenzoate-Mediated RAFT Polymerization: Status of dilemma. Macromol. Chem. Phys..

[61] Moad G., Rizzardo E., Thang S.H. (2012). Living Radical Polymerization by the RAFT Process—A Third Update. Aust. J. Chem..

[62] Moad G., Rizzardo E., Thang S.H. (2009). Living radical polymerization by the RAFT process A second update. Aust. J. Chem..

[63] Barner-Kowollik C., Buback M., Charleux B., Coote M.L., Drache M., Fukuda T., Goto A., Klumperman B., Lowe A.B., Mcleary J.B. (2006). Mechanism and Kinetics of Dithiobenzoate-Mediated RAFT Polymerization. I. The Current Situation. J. Polym. Sci. Part A Polym. Chem..

[64] Konkolewicz D., Hawkett B.S., Gray-Weale A., Perrier S. (2008). RAFT Polymerization Kinetics: Combination of Apparently Conflicting Models. Macromolecules.

[65] Kwak Y., Goto A., Tsujii Y., Murata Y., Komatsu K., Fukuda T. (2002). A Kinetic Study on the Rate Retardation in Radical Polymerization of Styrene with Addition−Fragmentation Chain Transfer. Macromolecules.

[66] Goto A., Sato K., Tsujii Y., Fukuda T., Moad G., Rizzardo E., Thang S.H. (2001). Mechanism and Kinetics of RAFT-Based Living Radical Polymerizations of Styrene and Methyl Methacrylate. Macromolecules.

[67] McLeary J.B., Calitz F.M., McKenzie J.M., Tonge M.P., Sanderson R.D., Klumperman B. (2004). Beyond Inhibition: A 1H NMR Investigation of the Early Kinetics of RAFT-Mediated Polymerization with the Same Initiating and Leaving Groups. Macromolecules.

[68] Calitz F.M., Tonge M.P., Sanderson R.D., Step D. (2006). Kinetic and Electron Spin Resonance Analysis of RAFT Polymerization of Styrene. Macromolecules.

[69] Geelen P., Klumperman B. (2007). Intermediate radical termination in reversible addition-fragmentation chain transfer-mediated polymerization: Identification of termination products. Macromolecules.

[70] Ranieri K., Delaittre G., Barner-Kowollik C., Junkers T. (2014). Direct access to dithiobenzonate RAFT agent fragmenting rate coefficients by ESR spin-trapping. Macromol. Rapid Commun..

[71] Kwak Y., Goto A., Fukuda T. (2004). Rate Retardation in Reversible Addition-Fragmentation Chain Transfer (RAFT) Polymerization: Further Evidence for Cross-Termination Producing 3-Arm Star Chain. Macromolecules.

[72] Meiser W., Buback M. (2011). Assessing the RAFT equilibrium constant via model systems: An EPR study-response to a comment. Macromol. Rapid Commun..

[73] Li C., He J., Liu Y., Zhou Y., Yang Y. (2012). Probing the RAFT process using a model reaction between alkoxyamine and dithioester. Aust. J. Chem..

[74] Konkolewicz D., Siauw M., Gray-Weale A., Hawkett B.S., Perrier S. (2009). Obtaining kinetic information from the chain-length distribution of polymers produced by RAFT. J. Phys. Chem. B.

[75] Ting S.R.S., Davis T.P., Zetterlund P.B. (2011). Retardation in RAFT polymerization: Does cross-termination occur with short radicals only?. Macromolecules.

[76] Junkers T., Delaittre G., Chapman R., Günzler F., Chernikova E., Barner-Kowollik C. (2012). Thioketone-mediated polymerization with dithiobenzoates: Proof for the existence of stable radical intermediates in RAFT polymerization. Macromol. Rapid Commun..

[77] Klumperman B., Van Den Dungen E.T.A., Heuts J.P.A., Monteiro M.J. (2010). RAFT-mediated polymerization-A story of incompatible data?. Macromol. Rapid Commun..

[78] Buback M., Vana P. (2006). Mechanism of Dithiobenzoate-Mediated RAFT Polymerization: A Missing Reaction Step. Macromol. Rapid Commun..

[79] Barner-Kowollik C., Coote M.L., Davis T.P., Radom L., Vana P. (2003). The reversible addition-fragmentation chain transfer process and the strength and limitations of modeling: Comment on “The magnitude of the fragmentation rate coefficient”. J. Polym. Sci. Part A Polym. Chem..

[80] Wang A.R., Zhu S., Kwak Y., Goto A., Fukuda T., Monteiro M.S. (2003). A difference of six orders of magnitude: A reply to “The magnitude of the fragmentation rate coefficient”. J. Polym. Sci. Part A Polym. Chem..

[81] Vana P., Davis T.P., Barner-Kowollik C. (2002). Kinetic analysis of reversible addition fragmentation chain transfer (RAFT) polymerizations: Conditions for inhibition, retardation, and optimum living polymerization. Macromol. Theory Simul..

[82] Zhang M., Ray W.H. (2001). Modeling of “living” free-radical polymerization with RAFT chemistry. Ind. Eng. Chem. Res..

[83] Monteiro M.J. (2005). Modeling the molecular weight distribution of block copolymer formation in a reversible addition-fragmentation chain transfer mediated living radical polymerization. J. Polym. Sci. Part A Polym. Chem..

[84] McLeary J.B., Tonge M.P., Klumperman B. (2006). A mechanistic interpretation of initialization processes in RAFT-mediated polymerization. Macromol. Rapid Commun..

[85] Drache M., Schmidt-Naake G., Buback M., Vana P. (2005). Modeling RAFT polymerization kinetics via Monte Carlo methods: Cumyl dithiobenzoate mediated methyl acrylate polymerization. Polymer (Guildf.).

[86] Coote M.L., Radom L. (2003). Ab initio evidence for slow fragmentation in RAFT polymerization. J. Am. Chem. Soc..

[87] Coote M.L. (2004). Ab initio study of the addition-fragmentation equilibrium in RAFT polymerization: When is polymerization retarded?. Macromolecules.

[88] Junkers T., Barner-Kowollik C., Coote M.L. (2011). Revealing model dependencies in “assessing the RAFT equilibrium constant via model systems: An EPR study”. Macromol. Rapid Commun..

[89] Junkers T. (2011). RAFT kinetics revisited: Revival of the RAFT debate. J. Polym. Sci. Part A Polym. Chem..

[90] Buback M., Meiser W., Vana P. (2009). Mechanism of CPDB-mediated RAFT polymerization of methyl methacrylate: Influence of pressure and RAFT agent concentration. Aust. J. Chem..

[91] Prescott S.W. (2003). Chain-length dependence in living/controlled free-radical polymerizations: Physical manifestation and Monte Carlo simulation of reversible transfer agents. Macromolecules.

[92] Tobita H. (2013). On the discrimination of RAFT models using miniemulsion polymerization. Macromol. Theory Simul..

[93] Tobita H. (2011). Effects of retardation and variation of monomer concentration in RAFT miniemulsion polymerization. Macromol. Theory Simul..

[94] Tobita H., Yanase F. (2007). Monte Carlo simulation of controlled/living radical polymerization in emulsified systems. Macromol. Theory Simul..

[95] Lansalot M., Davis T.P., Heuts J.P.A. (2002). RAFT miniemulsion polymerization: Influence of the structure of the RAFT agent. Macromolecules.

[96] Luo Y., Liu B., Wang Z., Gao J., Li B. (2007). Butyl Acrylate RAFT Polymerization in Miniemulsion. J. Polym. Sci. Part A Polym. Chem..

[97] Tsavalas J.G., Schork F.J., De Brouwer H., Monteiro M.J. (2001). Living Radical Polymerization by reversible addition-fragmentation chain transfer in ionically stabilized miniemulsions. Macromolecules.

[98] Devlaminck D.J.G., Van Steenberge P.H.M., Reyniers M.-F., D’hooge D.R. (2018). Deterministic Modeling of Degenerative RAFT Miniemulsion Polymerization Rate and Average Polymer Characteristics: Invalidity of Zero–One Nature at Higher Monomer Conversions. Macromolecules.

[99] Suzuki K., Kanematsu Y., Miura T., Minami M., Satoh S., Tobita H. (2014). Experimental method to discriminate RAFT models between intermediate termination and slow fragmentation via comparison of rates of miniemulsion and bulk polymerization. Macromol. Theory Simul..

[100] Tobita H. (2010). Fundamentals of RAFT miniemulsion polymerization kinetics. Macromol. Symp..

[101] Wang Z., Zhang Q., Zhan X., Chen F., Rao G., Xiong J. (2013). Preparation, kinetics and microstructures of well-defined PS-b-PS/Bd diblock copolymers via RAFT miniemulsion polymerization. J. Polym. Res..

[102] Suzuki K., Nishimura Y., Kanematsu Y., Masuda Y., Satoh S., Tobita H. (2012). Experimental Validation of Intermediate Termination in RAFT Polymerization with Dithiobenzoate via Comparison of Miniemulsion and Bulk Polymerization Rates. Macromol. React. Eng..

[103] Altarawneh I.S., Gomes V.G., Srour M.H. (2009). Polymer Chain Extension in Semibatch Emulsion Polymerization with RAFT-Based Transfer Agent: The influence of Reaction Conditions on Polymerization Rate and Product Properties. J. Appl. Polym. Sci..

[104] Altarawneh I.S., Gomes V.G., Srour M.S. (2008). The Influence of Xanthate-Based Transfer Agents on Styrene Emulsion Polymerization: Mathematical Modeling and Model Validation. Macromol. React. Eng..

[105] Luo Y., Wang R., Yang L., Yu B., Li B., Zhu S. (2006). Effect of Reversible Addition-Fragmentation Transfer (RAFT) reactions on (mini)emulsion polymerization kinetics and estimate of RAFT equilibrium constant. Macromolecules.

[106] Li X., Wang W.J., Weng F., Li B.G., Zhu S. (2014). Targeting copolymer composition distribution via model-based monomer feeding policy in semibatch RAFT mini-emulsion copolymerization of styrene and butyl acrylate. Ind. Eng. Chem. Res..

[107] Jung S.M., Gomes V.G. (2011). Miniemulsion polymerisation via reversible addition fragmentation chain transfer in pseudo-bulk regime. Macromol. React. Eng..

[108] Peklak A.D., Butte A. (2006). Kinetic Model of Reversible Addition Fragmentation Chain Transfer Polymerization of Styrene in Seeded Emulsion. J. Polym. Sci. Part A Polym. Chem..

[109] Smulders W., Gilbert R.G., Monteiro M.J. (2003). A kinetic investigation of seeded emulsion polymerization of styrene using reversible addition-fragmentation chain transfer (RAFT) agents with a low transfer constant. Macromolecules.

[110] Pepels M.P.F., Holdsworth C.I., Pascual S., Monteiro M.J. (2010). RAFT-Mediated emulsion polymerization of styrene with low reactive xanthate agents: Microemulsion-like behavior. Macromolecules.

[111] Butté A., Peklak A.D., Storti G., Morbidelli M. (2007). RAFT Polymerization in Bulk and Emulsion. Radic. Polym. Kinet. Mech..

[112] Luo Y., Yu B. (2005). Monte Carlo Simulation of Droplet Nucleation in RAFT Free Radical Miniemulsion Polymerization. Polym. Plast. Technol. Eng..

[113] Dossi M., Storti G., Moscatelli D. (2010). Initiation Kinetics in Free-Radical Polymerization: Prediction of Thermodynamic and Kinetic Parameters Based on ab initio Calculations. Macromol. Theory Simul..

[114] Buback M., Gilbert R.G., Hutchinson R.A., Klumberman B., Kuchta F.-D., Manders B.G., O’Driscoll K.F., Russell G.T., Schweer J. (1995). Critically evaluated rate coefficients for free-radical Propagation rate coefficient for styrene. Macromol. Chem. Phys..

[115] Johnston-Hall G., Monteiro M.J. (2008). Bimolecular Radical Termination: New Perspectives and Insights. J. Polym. Sci. Part A Polym. Chem..

[116] Derboven P., D’hooge D.R., Reyniers M.-F., Marin G.B., Barner-Kowollik C. (2015). The Long and the Short of Radical Polymerization. Macromolecules.

[117] Moad G., Solomon D.H. (2006). The Chemistry of Radical Polymerization.

[118] Wang A.R., Zhu S. (2003). Modeling the Reversible Addition–Fragmentation Transfer Polymerization Process. J. Polym. Sci. Part A Polym. Chem..

[119] Houshyar S., Keddie D.J., Moad G., Mulder R.J., Saubern S., Tsanaktsidis J. (2012). The scope for synthesis of macro-RAFT agents by sequential insertion of single monomer units. Polym. Chem..

[120] Van Steenberge P.H.M., D’hooge D.R., Reyniers M.F., Marin G.B., Cunningham M.F. (2014). 4-Dimensional modeling strategy for an improved understanding of miniemulsion NMP of acrylates initiated by SG1-macroinitiator. Macromolecules.

[121] Hui A.W., Hamielec A.E. (1972). Thermal Polymerization of Styrene at High Conversion and Temperatures. An Experimental Study. J. Appl. Polym. Sci..

[122] Van Steenberge P.H.M., D’hooge D.R., Wang Y., Zhong M., Reyniers M.-F., Konkolewicz D., Matyjaszewski K., Marin G.B. (2012). Linear Gradient Quality of ATRP Copolymers. Macromolecules.

[123] Bevington J.C., Melville H.W., Taylor R.P. (1954). The termination reaction in radical polymerizations. II. Polymerizations of styrene at 60° and of methyl methacrylate at 0 and 60°, and the copolymerization of these monomers at 60°. J. Polym. Sci..

[124] Fierens S.K., D’hooge D.R., Van Steenberge P.H.M., Reyniers M.-F., Marin G.B. (2014). MAMA-SG1 initiated nitroxide mediated polymerization of styrene: From Arrhenius parameters to model-based design. Chem. Eng. J..

[125] Khuong K.S., Jones W.H., Pryor W.A., Houk K.N. (2005). The mechanism of the self-initiated thermal polymerization of styrene. Theoretical solution of a classic problem. J. Am. Chem. Soc..

[126] Kotoulas C., Krallis A., Pladis P., Kiparissides C. (2003). A comprehensive kinetic model for the combined chemical and thermal polymerization of styrene up to high conversions. Macromol. Chem. Phys..

[127] Buback M., Barner-kowollik C., Kurz C., Wahl A. (2000). Termination kinetics of styrene free-radical polymerization studied by time-resolved pulsed laser experiments. Macromol. Chem. Phys..

[128] Vivaldo-Lima E., Mendoza-Fuentes A.D.J. (2002). Development of a kinetic model for INIFERTER controlled/“living” free-radical polymerization considering diffusion-controlled effects. Polym. React. Eng..

[129] Peklak A.D., Butté A., Storti G., Morbidelli M. (2006). Gel effect in the bulk reversible addition-fragmentation chain transfer polymerization of methyl methacrylate: Modeling and experiments. J. Polym. Sci. Part A Polym. Chem..

[130] Garg D.K., Serra C.A., Hoarau Y., Parida D., Bouquey M., Muller R. (2014). Analytical solution of free radical polymerization: Applications-implementing gel effect using AK model. Macromolecules.

[131] Tefera N., Weickert G., Westerterp K.R. (1996). Modeling of Free Radical Polymerization up to High Conversion. I. A Method for the Selection of Models by Simultaneous Parameter Estimation. J. Appl. Polym. Sci..

[132] Carswell T.G., Hill D.J.T., Londero D.I., O’Donnell J.H., Pomery P.J., Winzor C.L. (1992). Kinetic parameters for polymerization of methyl methacrylate at 60 °C. Polymer (Guildf.).

[133] Mastan E., Zhu S. (2015). Method of moments: A versatile tool for deterministic modeling of polymerization kinetics. Eur. Polym. J..

[134] D’hooge D.R., Reyniers M.-F., Marin G.B. (2009). Methodology for Kinetic Modeling of Atom Transfer Radical Polymerization. Macromol. React. Eng..

[135] Wang W., Zhou Y., Shi L., Luo Z.-H. (2014). Modeling of the Atom Transfer Radical Copolymerization Processes of Methyl Methacrylate and 2-(Trimethylsilyl) Ethyl Methacrylate under Batch, Semibatch, and Continuous Feeding: A Chemical Reactor Engineering Viewpoint. Ind. Eng. Chem. Res..

[136] Zhou Y.-N., Luo Z.-H. (2016). State-of-the-Art and Progress in Method of Moments for the Model-Based Reversible-Deactivation Radical Polymerization. Macromol. React. Eng..

[137] Smith W.V., Ewart R.H. (1948). Kinetics of emulsion polymerization. J. Chem. Phys..

[138] Zetterlund P.B., Okubo M. (2009). Compartmentalization in NMP in dispersed systems: Relative contributions of confined space effect and segregation effect depending on nitroxide type. Macromol. Theory Simul..

[139] Prescott S.W., Ballard M.J., Gilbert R.G. (2005). Average termination rate coefficients in emulsion polymerization: Effect of compartmentalization on free-radical lifetimes. J. Polym. Sci. Part A Polym. Chem..

[140] Jia Z., Monteiro M.J. (2012). Kinetic simulations of RAFT-mediated microemulsion polymerizations of styrene. ACS Symp. Ser..

[141] Zetterlund P.B. (2009). Nitroxide-Mediated Radical Polymerization in Dispersed Systems: Compartmentalization and Nitroxide Partitioning. Macromol. Theory Simul..

