# Supplementary material for: Modeling of Miniemulsion Polymerization of Styrene with Macro-RAFT Agents to Theoretically Compare Slow Fragmentation, Ideal Exchange and Cross-Termination Cases
Source: Polymers (Basel). 2019 Feb 13;11(2):320. doi: 10.3390/polym11020320 (PMC6419184; doi:10.3390/polym11020320)
Supplement: Supplementary file 1 [file polymers-11-00320-s001.pdf]

Supporting information for: Modeling of miniemulsion polymerization of styrene with macro-RAFT agents to theoretically compare slow fragmentation, ideal exchange and cross-termination cases

Dries J.G. Devlaminck[1], Paul H.M. Van Steenberge*[1], Marie-Françoise Reyniers[1], Dagmar R. D'hooge[1,2]

[1] Laboratory for Chemical Technology (LCT), Ghent University, Technologiepark 914, B-9052 Ghent Belgium
[2] Centre for Textile Science and Engineering, Ghent University, Technologiepark 907, B-9052 Ghent Belgium
* Correspondence: paul.vansteenberge@ugent.be;

Table of Contents

S1. Calculation of the average apparent termination rate coefficient 2

S2. Calculation of the apparent RAFT exchange related rate coefficients 3

S3. Prove of the insignificance of the RAFT cross termination rate coefficient for Comb3 (ideal RAFT agnet) 4

S4. Comparison between results of non-degenerative and degenerative model for Comb3 5

S5. Influence of diffusional limitations on the apparent rate coefficients for Comb1 6

S6. Influence of the (average) particle size on the average number of leaving group related radicals per particle 7

S7. Effect of average particle size on rates with the macro-RAFT intermediate radical. 8

S8. Population balances necessary to describe the temporal evolution of the polymer particles 8

S9. The continuity equations of the species in the aqueous phase, the abundant species in the particles and the associated equations for the average chain length characteristics 10

S1. Calculation of the average apparent termination rate coefficient

The diffusion-controlled mechanism of bimolecular termination in radical polymerization can be accurately described using the composite k_t model (RAFT-CLD-T model).[1] This model allows to calculate an apparent homotermination rate coefficient ($k_{t,app}^{i,i}$; i=chain length) dependent on the chain length i and the polymer mass fraction m_p (and thus the monomer conversion X_m):

For $i < i_{gel}$

$$k_{t,app}^{i,i} = k_t^{1,1}\, i^{-\alpha_s} \qquad \text{for } i < i_{SL} \tag{S1}$$

$$k_{t,app}^{i,i} = k_t^{1,1}\, i_{SL}^{(\alpha_L-\alpha_s)}\, i^{-\alpha_L} \qquad \text{for } i \geq i_{SL} \tag{S2}$$

For $i \geq i_{gel}$

$$k_{t,app}^{i,i} = k_t^{1,1}\, i_{gel}^{(\alpha_{gel}-\alpha_s)}\, i^{-\alpha_{gel}} \qquad \text{for } i < i_{SL} \tag{S3}$$

$$k_{t,app}^{i,i} = k_t^{1,1}\, i_{SL}^{(\alpha_L-\alpha_s)}\, i_{gel}^{(\alpha_{gel}-\alpha_L)}\, i^{-\alpha_{gel}} \qquad \text{for } i \geq i_{SL} \tag{S4}$$

in which $k_t^{1,1}$ is the (apparent) termination rate coefficient for radicals with chain length 1, α_s the exponent for termination for termination of short chains in dilute solution, α_L the exponent for long chains in dilute solution, α_{gel} the exponent for chains in the gel regime, i_{SL} the crossover chain length between short- and long-chain behavior, i_{gel} the chain length at the onset of the gel-effect.

An overview of these parameters can be found in Table S1.

Table S1: Parameters[1,2] used for the composite k_t model with styrene as monomer; m_p: polymer mass fraction

$k_t^{1,1}$	α_S	i_{SL}	α_L	α_{gel}	i_{gel}
10^9	0.53	30	0.15	$1.22m_p$-0.11	$3.30m_p^{-2.13}$

For simplicity, Equation (S1)-(S4) are calculated using the number average chain length of the macroradical species as the complete chain length distribution of the macroradicals is not calculated in the frame of the present work.

S2. Calculation of the apparent RAFT exchange related rate coefficients

The coupled parallel encounter pair model as described by D'hooge *et al.*[3] can be used to calculated the apparent rate coefficients for the RAFT addition, fragmentation, cross termination and transfer reactions involving only chain length *i* via:

$$k_{add,app}^{i,i} = \left(\frac{1}{k_{add,chem}} + \frac{1}{k_{add,diff}^{i,i}}\right)^{-1} \tag{S5}$$

$$k_{frag,app}^{i,i} = \left(\frac{1}{k_{frag,chem}} + \frac{K_{eq,1}}{k_{frag,diff}^{i,i}}\right)^{-1} \tag{S6}$$

$$k_{tcross,app}^{i,i,i} = \left(\frac{1}{k_{tcross,chem}} + \frac{1}{k_{tcross,diff}^{i,i,i}}\right)^{-1} \tag{S7}$$

$$k_{tr,0,app} = \left(\frac{1}{k_{tr,0,chem}} + \frac{1}{k_{tr,0,diff}} + \frac{1}{K_{eq,2}k_{-tr,0,diff}}\right)^{-1} \tag{S8}$$

$$k_{-tr,0,app} = \left(\frac{1}{k_{-tr,0,chem}} + \frac{1}{k_{-tr,0,diff}} + \frac{K_{eq,2}}{k_{tr,0,diff}}\right)^{-1} \tag{S9}$$

$$k_{tr,app} = \left(\frac{1}{k_{tr,chem}} + \frac{2}{k_{tr,diff}}\right)^{-1} \tag{S10}$$

with $k_{add,app}^{i,i}$, $k_{frag,app}^{i,i}$, $k_{tcross,app}^{i,i,i}$ and $k_{(-)tr(,0),app}^{i,i}$ the apparent RAFT addition, fragmentation, cross termination and transfer rate coefficient; $k_{add,chem}$, $k_{frag,chem}$, $k_{tcross,chem}$ and $k_{(-)tr(,0),chem}$ the intrinsic RAFT addition, fragmentation, cross termination and transfer rate coefficient (see Table 1 in the main text); $k_{add,diff}^{i,i}$, $k_{frag,diff}^{i,i}$, $k_{tcross,diff}^{i,i,i}$ and $k_{(-)tr(,0),diff}^{i,i}$ the diffusional contribution for RAFT addition, fragmentation, cross termination and transfer.

with:

$$K_{eq,1} = \frac{k_{add,chem}}{k_{frag,chem}} \tag{S11}$$

$$K_{eq,2} = \frac{k_{tr,0,chem}}{k_{-tr,0,chem}} \tag{S12}$$

In the present work, the diffusional contributions are approximated by the corresponding one when considering the composite k_t model[1] (see Section S1) for the apparent termination rate coefficient as this can also be described by the classical encounter pair theory:

$$k_{t,diff}^{i,i} \approx k_{add,diff}^{i,i} \approx k_{frag,diff}^{i,i} \approx k_{tcross,diff}^{i,i,i} \approx k_{(-)tr(,0),diff}^{i,i} \tag{S13}$$

in which the intrinsic termination rate coefficient ($k_{t,chem}$) is approximated by $k_t^{1,1}$ (see section S1). This approach is justified to a first approximation considering that the diffusion behavior of the species

involved in the RAFT reactions mentioned above is similar to the diffusion behavior of the corresponding terminating radicals.

Similar as for termination (Section S1), the number average chain length of the radicals are used to calculate the actual apparent rate coefficients.

S3. Prove of the insignificance of the RAFT cross termination rate coefficient for Comb3 (ideal RAFT agent)

As shown in Figure S1, the RAFT cross termination rate coefficient is kinetically insignificant for Comb3 as the same results are obtained when a value of 0 L mol^{-1} s^{-1} is used compared to a value of 10^8 L mol^{-1} s^{-1}.

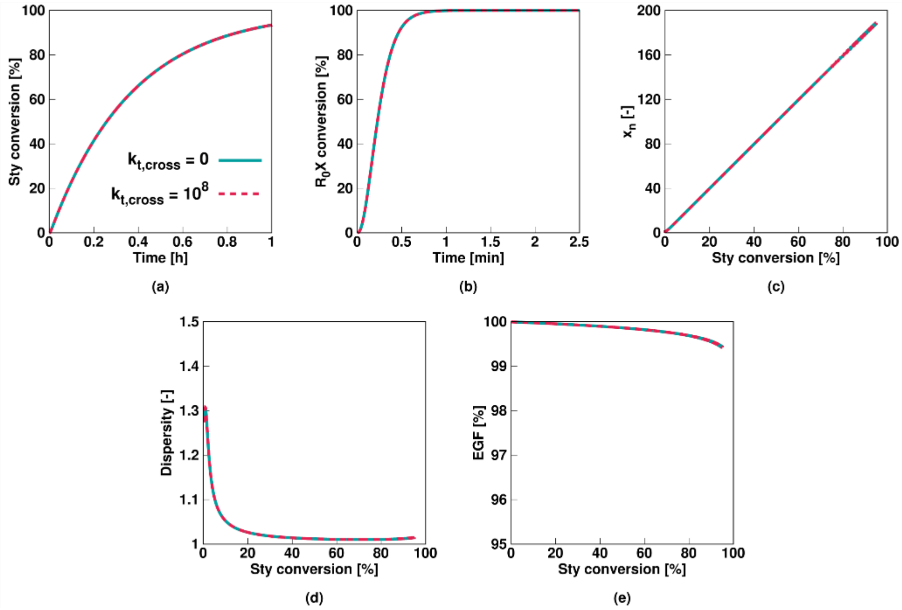

Figure S1: Overview of average characteristics for non-degenerative miniemulsion RAFT polymerization of styrene at 343 K with KPS and an oligomeric RAFT agent. Monomer conversion (a) and initial RAFT agent (R_0X) conversion (b) as a function of time and number average chain length x_n (c), dispersity (d) and end-group functionality (EGF) (e) as a function of styrene conversion. Model parameters for Comb3 in Table 1 without cross termination (blue) and with cross termination ($k_{t,cross}$ = 10^8 L mol^{-1} s^{-1}) taken into account. $[Sty]_0/[R_0X]_0 = 200$, $[R_0X]_0/[KPS]_0 = 3$, $[KPS]_0 = 4\ 10^{-3}$ mol L^{-1}, and $d_p = 100$ nm, $m_{styren,0} = 20$ g, $m_{H2O,0} = 80$ g

S4. Comparison between results of non-degenerative and degenerative model for Comb3

As shown in Figure S2, identical results are obtained when a non-degenerative or a degenerative model is used for Comb3. This implies that all intermediate species are very short-lived, as also can be seen in Figure 11 of the main text, and no side reactions involving these species are kinetically relevant (see also Section S3).

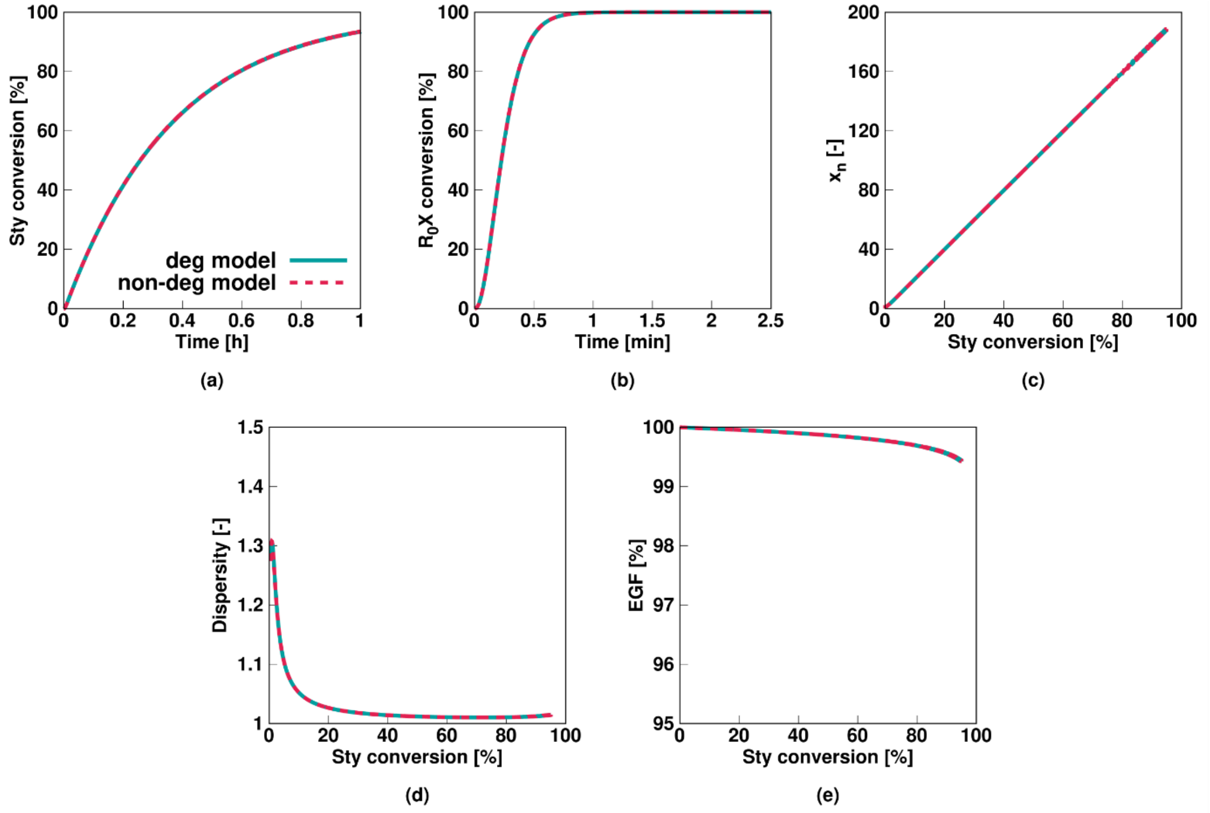

Figure S2: Overview of the degenerative (blue full line) and non-degenerative (dashed red line) simulation data for miniemulsion RAFT polymerization of styrene at 343 K with KPS and an oligomeric RAFT agent. Monomer conversion (a) and initial RAFT agent (R0X) conversion (b) as a function of time and number average chain length xn (c), dispersity (d) and EGF (d) as a function of styrene conversion. Model parameters for Comb3 in Table 1 of the main text. $[Sty]_0/[R_0X]_0$=200 , $[R_0X]_0/[KPS]_0$= 3 , $[KPS]_0$ = 4 10^{-3} mol L^{-1} , d_p = 100 nm, $m_{styren,0}$ = 20 g, $m_{H2O,0}$ = 80 g

S5. Influence of diffusional limitations on the apparent rate coefficients for Comb1

Figure S3 shows the influence of the diffusional limitations as taken into account via the formulas mentioned in Section S1 and S2 on the apparent rate coefficient for termination via recombination (a), addition of R_i to R_jX (b) and fragmentation of the intermediate radical to R_i and R_jX (c) for Comb1 (Table 1 of the main text).

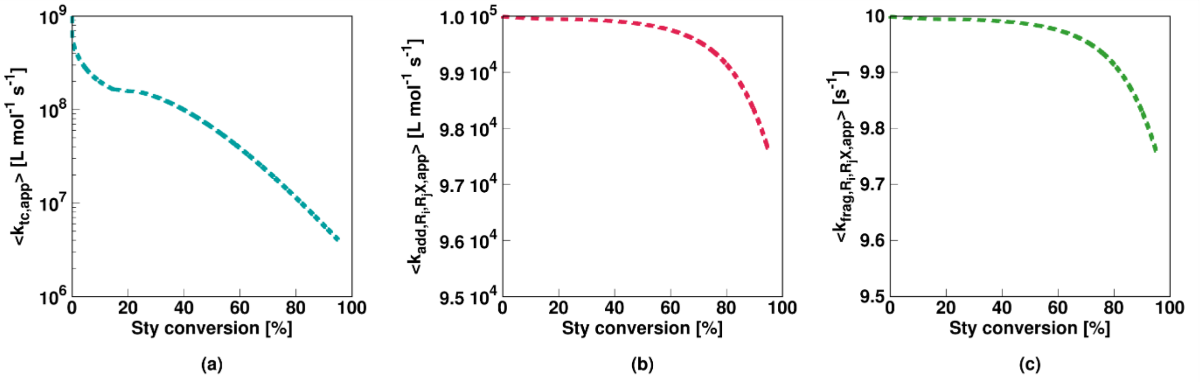

Figure S3: Overview of the apparent termination by recombination (a), addition of R_i to R_jX (b) and fragmentation to R_i to R_jX (c) rate coefficients as calculated by the formulas mentioned in Section S1 and S2 for miniemulsion RAFT polymerization of styrene at 343 K with KPS and an oligomeric RAFT agent. Model parameters for Comb1 in Table 1 of the main text. $[Sty]_0/[R_0X]_0$=200 , $[R_0X]_0/[KPS]_0$= 3 , $[KPS]_0$ = 4 10^{-3} mol L^{-1} , d_p = 100 nm , $m_{styrene,0}$ = 20 g, $m_{H2O,0}$ = 80 g; note no RAFT cross-termination.

S6. Influence of the (average) particle size on the average number of leaving group related radicals per particle

Figure S4 shows the influence of the (average) particle size on average number of leaving group (blue), R_0XR_i (green) and R_0XR_0 (purple) radicals per particle for model parameters Comb1 (first column), Comb2 (second column) and Comb3 (third column) at 30% monomer conversion. At this monomer conversion, the conversion of initial RAFT agent (R_0X) is 100% resulting in a negligible amount of these leaving group related radicals. For a more in depth investigation of the influence of the (average) particle size on the three investigated model parameter sets, the reader is referred to the main text.

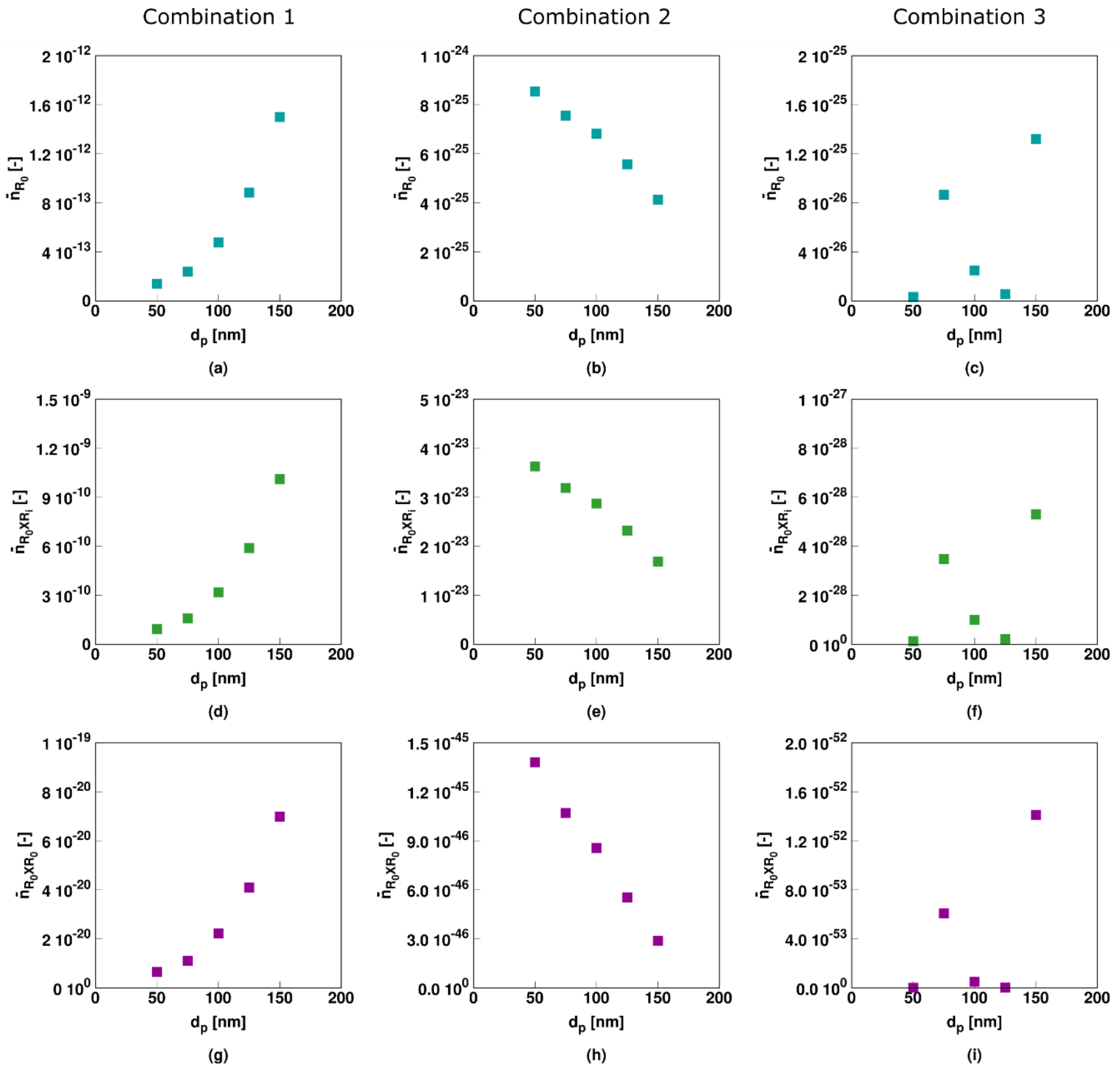

Figure S4: Influence of the (average) particle size (dp) on the average number of leaving group ((a)-(c), blue), R_0XR_i ((d)-(f), green) and R_0XR_0 ((g)-(i), purple) radicals per particle for model parameters Comb1 (first column), Comb2 (second column) and Comb3 (third column) in Table 1. [Sty]0/[R0X]0=200 , [R0X]0/[KPS]0= 3 , [KPS]0 = 4 10-3 mol L-1 , mstyrene,0 = 20 g, mH2O,0 = 80 g. XM is 30% and XR0X is 100% for all simulation points.

S7. Effect of average particle size on rates with the macro-RAFT intermediate radical.

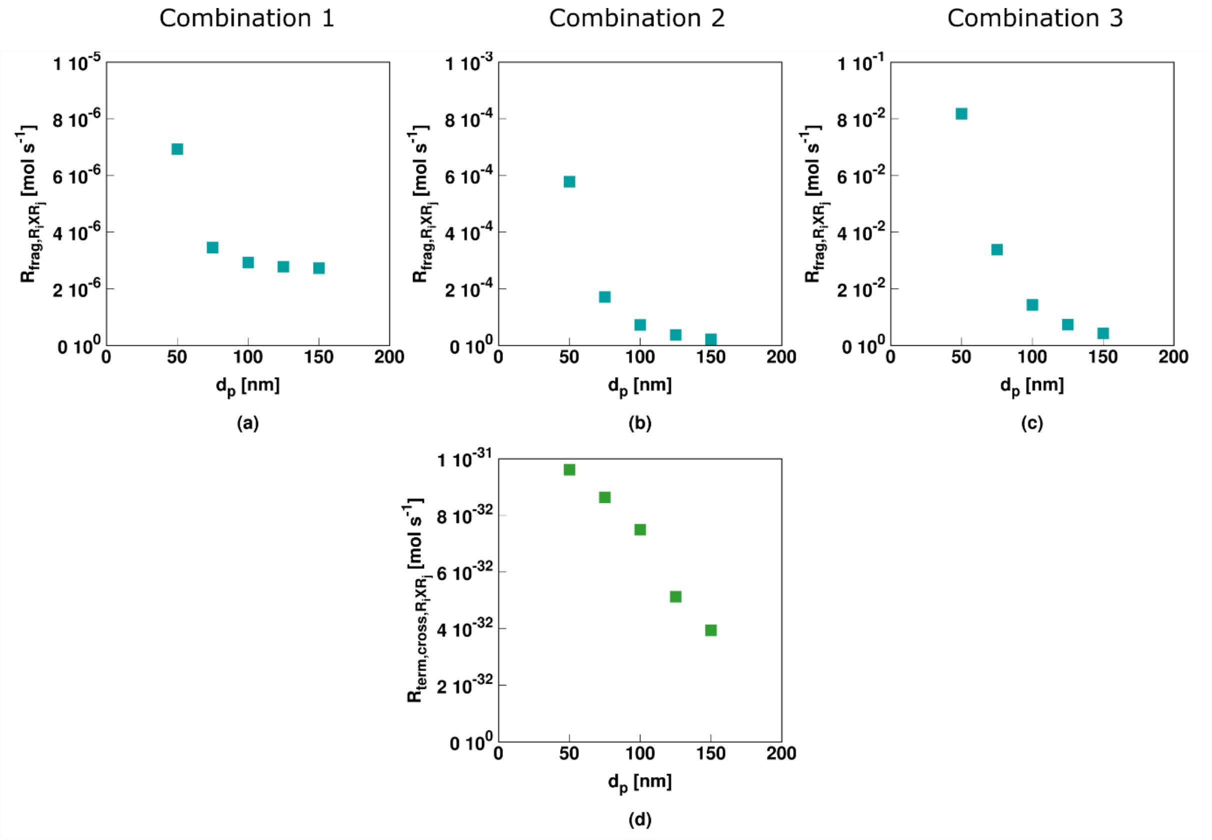

Figure S5: Fragmentation and RAFT cross-termination rates for R_iXR_j radicals related to Figure 14 in the main text.

Figure S5 shows the corresponding rates for Figure 14 in the main text, taking the macro-RAFT intermediate radical as reference. It specifically follows that for Comb1 at the higher d_p a bulk-like character is is obtained. Notably for Comb 3 the RAFT fragmentation rates are the highest. Furthermore, in Comb2, the RAFT cross-termination rate is the lowest at the highest d_p but relatively the importance of RAFT-cross termination is higher (less segregation).

S8. Population balances necessary to describe the temporal evolution of the polymer particles

The population balances of the five-dimensional Smith-Ewart model as necessary for the non-degenerative model (Table 1), describing the temporal evolution of polymer particles having k macroradicals, l R_0 radicals, m R_iXR_0 radicals, n R_iXR_j radicals and o R_0XR_0 radicals ($N_{k,l,m,n,o}$; $k,l,m,n,o \geq 0$) are given by:

$$\frac{dN_{k,l,m,n,o}}{dt} = \frac{k_{tc,app}}{2v_pN_A}\Big((k+2)(k+1)N_{k+2,l,m,n,o} - k(k-1)N_{k,l,m,n,o}\Big) + k_{entryR_2}\big[R_{2,aq}\big](N_{k-1,l,m,n,o}$$
$$- N_{k,l,m,n,o}) + k_{pR_0}\big[M_p\big]\Big((l+1)N_{k-1,l+1,m,n,o} - lN_{k,l,m,n,o}\Big)$$
$$+ \frac{k_{tc,00}}{2v_pN_A}\Big((l+2)(l+1)N_{k,l+2,m,n,o} - l(l-1)N_{k,l,m,n,o}\Big)$$
$$+ \frac{k_{tc,0}}{v_pN_A}\Big((k+1)(l+1)N_{k+1,l+1,m,n,o} - klN_{k,l,m,n,o}\Big)$$
$$+ k_{add,R_0,R_iX}\big[R_iX_p\big]\Big((l+1)N_{k,l+1,m-1,n,o} - lN_{k,l,m,n,o}\Big)$$
$$+ k_{add,R_i,R_jX}\big[R_jX_p\big]\Big((k+1)N_{k+1,l,m,n-1,o} - kN_{k,l,m,n,o}\Big)$$
$$+ k_{add,R_0,R_0X}\big[R_0X_p\big]\Big((l+1)N_{k,l+1,m,n,o-1} - lN_{k,l,m,n,o}\Big)$$
$$+ k_{add,R_i,R_0X}\big[R_0X_p\big]\Big((k+1)N_{k+1,l,m-1,n,o} - kN_{k,l,m,n,o}\Big)$$
$$+ k_{frag,R_0,R_iX}\Big((m+1)N_{k,l-1,m+1,n,o} - mN_{k,l,m,n,o}\Big)$$
$$+ k_{frag,R_i,R_0X}\Big((m+1)N_{k-1,l,m+1,n,o} - mN_{k,l,m,n,o}\Big) \tag{S14}$$
$$+ k_{frag,R_i,R_jX}\Big((n+1)N_{k-1,l,m,n+1,o} - nN_{k,l,m,n,o}\Big)$$
$$+ k_{frag,R_0,R_0X}\Big((o+1)N_{k,l-1,m,n,o+1} - oN_{k,l,m,n,o}\Big)$$
$$+ \frac{k_{tcross}}{v_pN_A}\Big((k+1)(m+1)N_{k+1,l,m+1,n,o} - kmN_{k,l,m,n,o}\Big)$$
$$+ \frac{k_{tcross}}{v_pN_A}\Big((l+1)(m+1)N_{k,l+1,m+1,n,o} - lmN_{k,l,m,n,o}\Big)$$
$$+ \frac{k_{tcross}}{v_pN_A}\Big((k+1)(n+1)N_{k+1,l,m,n+1,o} - knN_{k,l,m,n,o}\Big)$$
$$+ \frac{k_{tcross}}{v_pN_A}\Big((l+1)(n+1)N_{k,l+1,m,n+1,o} - lnN_{k,l,m,n,o}\Big)$$
$$+ \frac{k_{tcross}}{v_pN_A}\Big((k+1)(o+1)N_{k+1,l,m,n,o+1} - koN_{k,l,m,n,o}\Big)$$
$$+ \frac{k_{tcross}}{v_pN_A}\Big((l+1)(o+1)N_{k,l+1,m,n,o+1} - loN_{k,l,m,n,o}\Big)$$

S9. The continuity equations of the species in the aqueous phase, the abundant species in the particles and the associated equations for the average chain length characteristics

The continuity equations of the species in the aqueous phase, the abundant species in the particles and the pseudo-bulk approximation of the higher order moment equations of the macrospecies are given by:

$$\frac{d[I_{2,aq}]}{dt} = -k_{dis}[I_{2,aq}] \tag{S15}$$

$$\frac{d[I_{aq}]}{dt} = 2fk_{dis}[I_{2,aq}] - k_{pI}[I_{aq}][M_{aq}] \tag{S16}$$

$$\frac{d[R_{1,aq}]}{dt} = k_{pI}[I_{aq}][M_{aq}] - k_p[R_{1,aq}][M_{aq}] \tag{S17}$$

$$\frac{d[R_{2,aq}]}{dt} = k_p[R_{1,aq}][M_{aq}] - k_{EntryR2}N_p\frac{[R_{2,aq}]}{N_AV_{aq}} \tag{S18}$$

$$\frac{d[M_{aq}]}{dt} = -k_{pI}[I_{aq}][M_{aq}] - k_p[R_{1,aq}][M_{aq}] \tag{S19}$$

$$\frac{d[M_p]}{dt} = -k_p\frac{\bar{n}(R_i)}{N_a v_p}[M_p] - k_{pR_0}\frac{\bar{n}(R_0)}{N_a v_p}[M_p] \tag{S20}$$

$$\begin{aligned}\frac{d\tau_0}{dt} = \frac{d\sum_{i>0}[R_iX_p]}{dt} &= k_{frag,R_0,R_iX}\frac{\bar{n}(R_0XR_i)}{N_a v_p} + k_{frag,R_i,R_jX}\frac{\bar{n}(R_iXR_j)}{N_a v_p} \\ &- k_{add,R_0,R_iX}\frac{\bar{n}(R_0)}{N_a v_p}\tau_0 - k_{add,R_i,R_jX}\frac{\bar{n}(R_i)}{N_a v_p}\tau_0\end{aligned} \tag{S21}$$

$$\begin{aligned}\frac{d\mu_0}{dt} = \frac{d\sum_{i\geq 1}[P_{i,p}]}{dt} &= \frac{k_{tc,app}}{2}\frac{\bar{n}(R_i)}{N_a v_p}\frac{\bar{n}(R_i)}{N_a v_p} + k_{tc,0}\frac{\bar{n}(R_i)}{N_a v_p}\frac{\bar{n}(R_0)}{N_a v_p} + k_{tcross}\frac{\bar{n}(R_i)}{N_a v_p}\frac{\bar{n}(R_0XR_i)}{N_a v_p} \\ &+ k_{tcross}\frac{\bar{n}(R_0)}{N_a v_p}\frac{\bar{n}(R_0XR_i)}{N_a v_p} + k_{tcross}\frac{\bar{n}(R_i)}{N_a v_p}\frac{\bar{n}(R_iXR_j)}{N_a v_p} \\ &+ k_{tcross}\frac{\bar{n}(R_0)}{N_a v_p}\frac{\bar{n}(R_iXR_j)}{N_a v_p} + k_{tcross}\frac{\bar{n}(R_i)}{N_a v_p}\frac{\bar{n}(R_0XR_0)}{N_a v_p}\end{aligned} \tag{S22}$$

$$\frac{d[P_{0,p}]}{dt} = \frac{k_{tc,00}}{2}\frac{\bar{n}(R_0)}{N_a v_p}\frac{\bar{n}(R_0)}{N_a v_p} + k_{tcross}\frac{\bar{n}(R_0)}{N_a v_p}\frac{\bar{n}(R_0XR_0)}{N_a v_p} \tag{S23}$$

$$\begin{aligned}\frac{d[R_0X_p]}{dt} &= k_{frag,R_i,R_0X}\frac{\bar{n}(R_0XR_i)}{N_a v_p} + k_{frag,R_0,R_0X}\frac{\bar{n}(R_0XR_0)}{N_a v_p} \\ &- k_{add,R_0,R_0X}\frac{\bar{n}(R_0)}{N_a v_p}[R_0X_p] - k_{add,R_i,R_0X}\frac{\bar{n}(R_i)}{N_a v_p}[R_0X_p]\end{aligned} \tag{S24}$$

$$\frac{d\lambda_1}{dt}=\frac{d\sum_i i[R_i]}{dt} \tag{S25}$$
$$=[M_p]\left(k_p\frac{\overline{n}(R_i)}{N_a v_p}+k_{pR_0}\frac{\overline{n}(R_0)}{N_a v_p}\right)+2k_{entryR_2}N_p\frac{[R_{2,aq}]}{N_A V_{aq}}$$
$$-k_{tc,app,c}\frac{\overline{n}(R_i)}{N_a v_p}\lambda_1-k_{tc,0,c}\lambda_1\frac{\overline{n}(R_0)}{N_a v_p}-k_{add,R_i,R_0X}\lambda_1[R_0X_p]$$
$$-k_{add,R_i,R_jX,c}\lambda_1\tau_0+k_{frag,R_i,R_0X}\Omega_1^0+k_{frag,R_i,R_jX}\Omega_{1,0}$$
$$-k_{tcross,c}\lambda_1\frac{\overline{n}(R_0XR_0)}{N_a v_p}-k_{tcross,c}\lambda_1\frac{\overline{n}(R_0XR_i)}{N_a v_p}-k_{tcross,c}\lambda_1\frac{\overline{n}(R_iXR_j)}{N_a v_p}$$

$$\frac{d\lambda_2}{dt}=\frac{d\sum_i i^2[R_i]}{dt} \tag{S26}$$
$$=[M_p]\left(k_p\frac{\overline{n}(R_i)}{N_a v_p}+2k_p\lambda_1+k_{pR_0}\frac{\overline{n}(R_0)}{N_a v_p}\right)+4k_{entryR_4}N_p\frac{[R_{2,aq}]}{N_A V_{aq}}$$
$$-k_{tc,app,c}\frac{\overline{n}(R_i)}{N_a v_p}\lambda_2-k_{tc,0,c}\lambda_2\frac{\overline{n}(R_0)}{N_a v_p}-k_{add,R_i,R_0X}\lambda_2[R_0X_p]$$
$$-k_{add,R_i,R_jX,c}\lambda_2\tau_0+k_{frag,R_i,R_0X}\Omega_2^0+k_{frag,R_i,R_jX}\Omega_{2,0}$$
$$-k_{tcross,c}\lambda_2\frac{\overline{n}(R_0XR_0)}{N_a v_p}-k_{tcross,c}\lambda_2\frac{\overline{n}(R_0XR_i)}{N_a v_p}-k_{tcross,c}\lambda_2\frac{\overline{n}(R_iXR_j)}{N_a v_p}$$

$$\frac{d\tau_1}{dt}=\frac{d\sum_i i[R_iX_p]}{dt} \tag{S27}$$
$$=k_{frag,R_0,R_iX}\Omega_1^0+k_{frag,R_i,R_jX}\Omega_{1,0}-k_{add,R_0,R_iX}\frac{\overline{n}(R_0)}{N_a v_p}\tau_1$$
$$-k_{add,R_i,R_jX,c}\frac{\overline{n}(R_i)}{N_a v_p}\tau_1$$

$$\frac{d\tau_2}{dt}=\frac{d\sum_i i^2[R_iX_p]}{dt} \tag{S28}$$
$$=k_{frag,R_0,R_iX}\Omega_2^0+k_{frag,R_i,R_jX}\Omega_{2,0}-k_{add,R_0,R_iX}\frac{\overline{n}(R_0)}{N_a v_p}\tau_2$$
$$-k_{add,R_i,R_jX,c}\frac{\overline{n}(R_i)}{N_a v_p}\tau_2$$

$$\frac{d\mu_1}{dt}=\frac{d\sum_{i\geq 1} i[P_{i,p}]}{dt} \tag{S29}$$
$$=k_{tc,app,c}\frac{\overline{n}(R_i)}{N_a v_p}\lambda_1+k_{tc,0,c}\lambda_1\frac{\overline{n}(R_0)}{N_a v_p}+k_{tcross,c}\Omega_1^0\frac{\overline{n}(R_i)}{N_a v_p}$$
$$+k_{tcross,c}\Omega_{1,0}\frac{\overline{n}(R_i)}{N_a v_p}+k_{tcross,c}\lambda_1\frac{\overline{n}(R_0XR_0)}{N_a v_p}+k_{tcross,c}\Omega_1^0\frac{\overline{n}(R_0)}{N_a v_p}$$
$$+k_{tcross,c}\Omega_{1,0}\frac{\overline{n}(R_0)}{N_a v_p}$$

$$\frac{d\mu_2}{dt}=\frac{d\sum_{i\geq 1} i^2[P_{i,p}]}{dt} \tag{S30}$$
$$=k_{tc,app,c}\frac{\overline{n}(R_i)}{N_a v_p}\lambda_2+k_{tc,0,c}\lambda_2\frac{\overline{n}(R_0)}{N_a v_p}+k_{tcross,c}\Omega_2^0\frac{\overline{n}(R_i)}{N_a v_p}$$
$$+k_{tcross,c}\Omega_{2,0}\frac{\overline{n}(R_i)}{N_a v_p}+k_{tcross,c}\lambda_2\frac{\overline{n}(R_0XR_0)}{N_a v_p}+k_{tcross,c}\Omega_2^0\frac{\overline{n}(R_0)}{N_a v_p}$$
$$+k_{tcross,c}\Omega_{2,0}\frac{\overline{n}(R_0)}{N_a v_p}$$

$$\frac{d\Omega_1^0}{dt} = \frac{d\sum_{i\geq 1} i[R_0XR_{i,p}]}{dt}$$
$$= k_{add,R_0,R_iX}\frac{\overline{n}(R_0)}{N_a v_p}\tau_1 + k_{add,R_i,R_0X}\lambda_1[R_0X_p] - k_{frag,R_i,R_0X}\Omega_1^0$$
$$- k_{frag,R_0,R_iX}\Omega_1^0 - k_{tcross,c}\Omega_1^0\frac{\overline{n}(R_i)}{N_a v_p} - k_{tcross,c}\Omega_1^0\frac{\overline{n}(R_0)}{N_a v_p} \quad \text{(S31)}$$

$$\frac{d\Omega_2^0}{dt} = \frac{d\sum_{i\geq 1} i^2[R_0XR_{i,p}]}{dt}$$
$$= k_{add,R_0,R_iX}\frac{\overline{n}(R_0)}{N_a v_p}\tau_2 + k_{add,R_i,R_0X}\lambda_2[R_0X_p] - k_{frag,R_i,R_0X}\Omega_2^0$$
$$- k_{frag,R_0,R_iX}\Omega_2^0 - k_{tcross,c}\Omega_2^0\frac{\overline{n}(R_i)}{N_a v_p} - k_{tcross,c}\Omega_2^0\frac{\overline{n}(R_0)}{N_a v_p} \quad \text{(S32)}$$

$$\frac{d\Omega_{1,0}}{dt} = \frac{d\sum_{i\geq 1}\sum_{j\geq 1} i[R_iXR_j]}{dt}$$
$$= k_{add,R_i,R_jX,c}\left(\lambda_1\tau_0 + \frac{\overline{n}(R_i)}{N_a v_p}\tau_1\right) - 2k_{frag,R_i,R_jX}\Omega_{1,0}$$
$$- k_{tcross,c}\Omega_{1,0}\frac{\overline{n}(R_i)}{N_a v_p} - k_{tcross,c}\Omega_{1,0}\frac{\overline{n}(R_0)}{N_a v_p} \quad \text{(S33)}$$

$$\frac{d\Omega_{2,0}}{dt} = \frac{d\sum_{i\geq 1}\sum_{j\geq 1} i^2[R_iXR_j]}{dt}$$
$$= k_{add,R_i,R_jX,c}\left(\lambda_2\tau_0 + \frac{\overline{n}(R_i)}{N_a v_p}\tau_2\right) - 2k_{frag,R_i,R_jX}\Omega_{2,0}$$
$$- k_{tcross,c}\Omega_{2,0}\frac{\overline{n}(R_i)}{N_a v_p} - k_{tcross,c}\Omega_{2,0}\frac{\overline{n}(R_0)}{N_a v_p} \quad \text{(S34)}$$

$$\frac{d\Omega_{1,1}}{dt} = \frac{d\sum_{i\geq 1}\sum_{j\geq 1} ij[R_iXR_j]}{dt}$$
$$= 2k_{add,R_i,R_jX,c}\lambda_1\tau_1 - 2k_{frag,R_i,R_jX}\Omega_{1,1} - k_{tcross,c}\Omega_{1,1}\frac{\overline{n}(R_i)}{N_a v_p}$$
$$- k_{tcross,c}\Omega_{1,1}\frac{\overline{n}(R_0)}{N_a v_p} \quad \text{(S35)}$$

with the number and mass average chain length of the R_iXR_j radicals defined as

$$x_{n,R_iXR_j} = \frac{\Omega_{1,0}}{\frac{\overline{n}(R_iXR_j)}{N_a v_p}} \quad \text{(S36)}$$

$$x_{m,RiXR_j} = \frac{\Omega_{2,0} + \Omega_{1,1}}{\Omega_{1,0}} \quad \text{(S37)}$$

References:

1. Johnston-Hall, G.; Monteiro, M.J. Bimolecular Radical Termination: New Perspectives and Insights. *J. Polym. Sci. Part a-Polymer Chem.* **2008**, *46*, 3155–3176.

2. Derboven, P.; D’hooge, D.R.; Reyniers, M.-F.; Marin, G.B.; Barner-Kowollik, C. The Long and the Short of Radical Polymerization. *Macromolecules* **2015**, *48*, 492–501.

3. D’hooge, D.R.; Reyniers, M.F.; Marin, G.B. The crucial role of diffusional limitations in controlled radical polymerization. *Macromol. React. Eng.* **2013**, *7*, 362–379.
